# The evolution of plant proton pump regulation via the R domain may have facilitated plant terrestrialization

**DOI:** 10.1038/s42003-022-04291-y

**Published:** 2022-11-29

**Authors:** Anett Stéger, Maki Hayashi, Emil Wacenius Lauritzen, Klaus Herburger, Lana Shabala, Cuiwei Wang, Amalie Kofoed Bendtsen, Anton Frisgaard Nørrevang, Kenneth Madriz-Ordeñana, Shichao Ren, Mai Duy Luu Trinh, Hans Thordal‑Christensen, Anja Thoe Fuglsang, Sergey Shabala, Jeppe Thulin Østerberg, Michael Palmgren

**Affiliations:** 1 Department of Plant and Environmental Sciences, University of Copenhagen, Thorvaldsensvej 40, DK-1871 Frederiksberg C, Denmark; 2 Tasmanian Institute of Agriculture, University of Tasmania, Hobart, TAS 7001 Australia; 3 International Research Centre for Environmental Membrane Biology, Foshan University, 528000 Foshan, China

**Keywords:** Plant evolution, Plant transporters

## Abstract

Plasma membrane (PM) H^+^-ATPases are the electrogenic proton pumps that export H^+^ from plant and fungal cells to acidify the surroundings and generate a membrane potential. Plant PM H^+^-ATPases are equipped with a C‑terminal autoinhibitory regulatory (R) domain of about 100 amino acid residues, which could not be identified in the PM H^+^-ATPases of green algae but appeared fully developed in immediate streptophyte algal predecessors of land plants. To explore the physiological significance of this domain, we created in vivo C-terminal truncations of autoinhibited PM H^+^‑ATPase2 (AHA2), one of the two major isoforms in the land plant *Arabidopsis thaliana*. As more residues were deleted, the mutant plants became progressively more efficient in proton extrusion, concomitant with increased expansion growth and nutrient uptake. However, as the hyperactivated AHA2 also contributed to stomatal pore opening, which provides an exit pathway for water and an entrance pathway for pests, the mutant plants were more susceptible to biotic and abiotic stresses, pathogen invasion and water loss, respectively. Taken together, our results demonstrate that pump regulation through the R domain is crucial for land plant fitness and by controlling growth and nutrient uptake might have been necessary already for the successful water-to-land transition of plants.

## Abstract (web summary)

Phylogenetic analyses and physiological experiments indicate that the regulatory domain of plant proton pumps could have been key in the plant transition to land from water.

## Introduction

The plant plasma membrane (PM) H^+^-ATPase is a master enzyme that serves as a H^+^ pump to generate an electrochemical gradient of H^+^ across the PM, which in turn energizes the transport of inorganic ions, metabolites, and water in and out of the cell through other transport systems^1,2^. The plant cell is enveloped by a rigid supporting cell wall, which is softened when acidified, and the acid growth hypothesis proposes that plant growth hormones, such as auxin facilitate cell expansion growth by activating H^+^ extrusion by the PM H^+^-ATPase^3^. Recent studies demonstrated that overexpression of genes encoding PM H^+^‑ATPases promotes plant growth apparently without loss of fitness^4,5^. A major effect of this overexpression is stimulation of the hydraulic mechanism that drives the opening of the stomatal pores that facilitate CO_2_ uptake. Improved stomatal conductance leads to increased photosynthetic rates and results in increased biomass; for this reason, the potential of PM H^+^-ATPase to increase plant production via genetic modification has been proposed^6^.

Eleven isoforms of PM H^+^-ATPases, termed AUTOINHIBITED H^+^-ATPASE 1–11 (AHA1–11), are encoded in the genome of the model plant *Arabidopsis thaliana*^7^. Among the 11 gene family members, *AHA1* and *AHA2* are the most highly expressed^8^. *AHA1* is expressed mainly in photosynthetic tissues^8,9^, while *AHA2* is predominantly expressed in roots and root hairs^8,10^. AHA1 and AHA2 are closely related and likely arose from a recent duplication of a common ancestor.

As a key enzyme, the PM H^+^-ATPase is tightly regulated by multiple internal and external stimuli, including phytohormones, light, nutrient status, pH, and molecular signatures of pathogens^11^. Most of the regulation proceeds via a C-terminal autoinhibitory regulatory (R) domain of about 100 amino acid residues^12,13^. In a fungal PM H^+^-ATPase, the corresponding but much shorter region connects the pump to a neighboring PM H^+^-ATPase in a hexameric ring to generate a rigid low‑activity complex^14,15^. In the available structure of a plant PM H^+^-ATPase, the R domain is not resolved^16^, but cross-linking experiments indicate that it interacts with the catalytic domain of the neighbor PM H^+^-ATPase in a dimer^17^.

The R domain of plant PM H^+^-ATPase includes two autoinhibitory regions, Region I and Region II^18^, and is a target of multiple proteins and lipids that affect the strength of the interaction between the R domain and the rest of the pump molecule^2^. A major mechanism for activation of the plant PM H^+^-ATPase proceeds via protein kinase-mediated phosphorylation of the penultimate residue, a threonine (Thr-947 in AHA2), that is downstream of both autoinhibitory regions^19^. This creates a binding site for 14-3-3 protein, and its subsequent binding relieves the negative effect of both autoinhibitory regions^20^. Several protein kinases have Thr-947 as a target, including transmembrane kinases (TMKs), which are activated in response to the plant growth hormone auxin^21,22^. Phosphorylation of a nearby Ser residue (Ser-932 in AHA2) prevents the binding of 14-3-3 protein regardless of whether Thr-947 is phosphorylated or not^23^. Phosphorylation of Thr-881 in Region I causes 14-3-3 protein-independent activation of the pump^24^. More phosphosites have been identified in the R domain, and their phosphorylation status is also regulated by protein phosphatases, including PP2C.D, which acts on phosphorylated Thr-947. Auxin-induced SAUR proteins inhibit PP2C.D with pump activation as the result^25,26^.

Based on our phylogenetic study, the plant R domain is unique for land plants and some streptophyte algae, the closest ancestors of land plants. This would suggest that the evolution of the plant R domain may have provided an advantage for the water-to-land transition of plants. To test this hypothesis, we analyzed the physiological consequences of progressively neutralizing the R domain of AHA2 by CRISPR/Cas9-induced truncations at the gene level. Phenotypic analysis of these mutants provides important information with respect to the physiological role of AHA2 and to the importance of the R domain for possibly other land plants.

## Results

### The R domain of plant PM H^+^-ATPase evolved in streptophyte algae

**Fig. 1:**
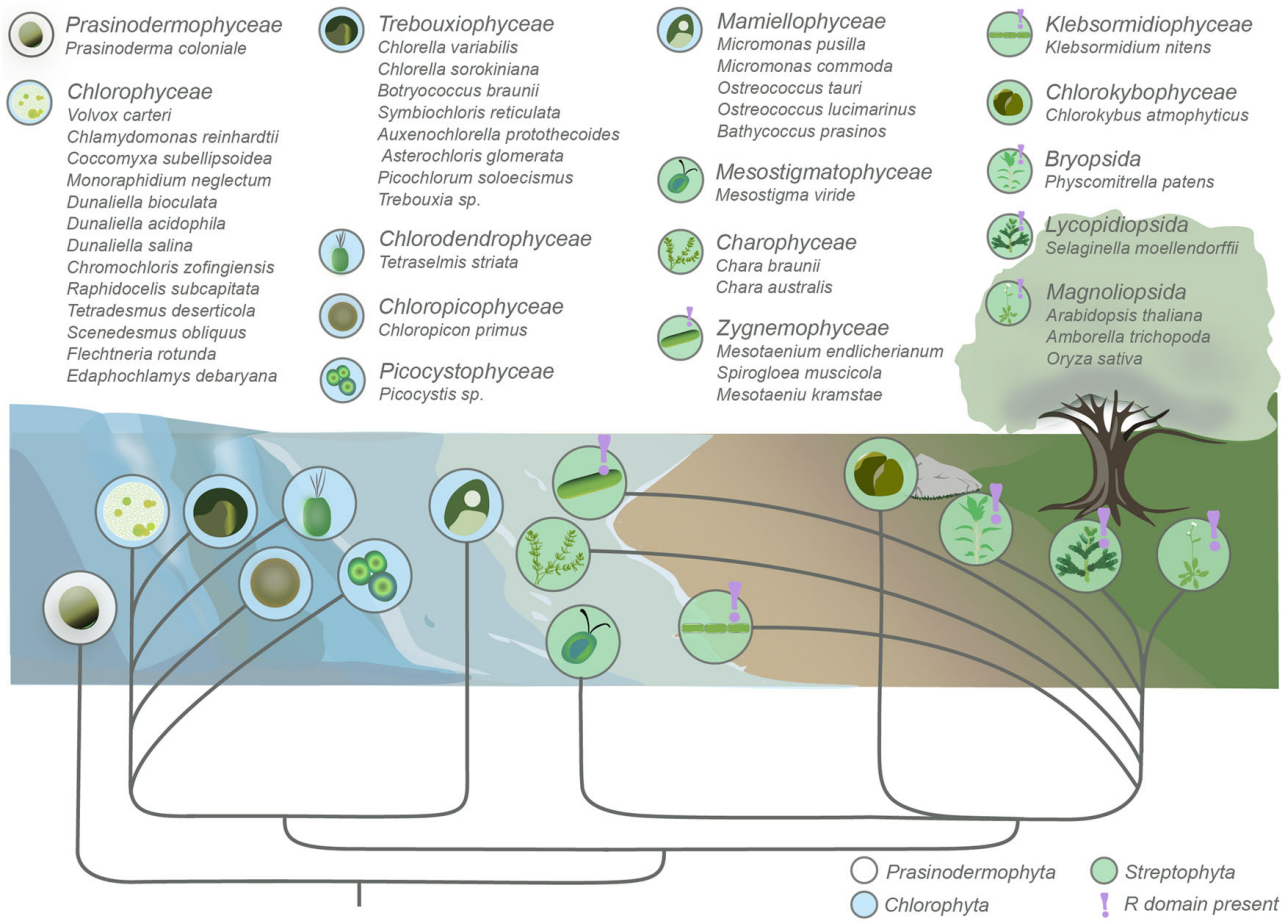
The R domain of the plant plasma membrane H^+^-ATPase appeared in the immediate streptophyte predecessors of land plants. The figure shows the analyzed representative species within different classes from Prasinodermophyta, Chlorophyta, and Streptophyta. The drawing illustrates the most typical habitat of the different classes: water, water and soil or soil. Purple exclamation marks indicate the presence of the plant PM H^+^-ATPase R domain. The figure is adapted from Cheng et al. (2019)^60^.

**Fig. 2:**
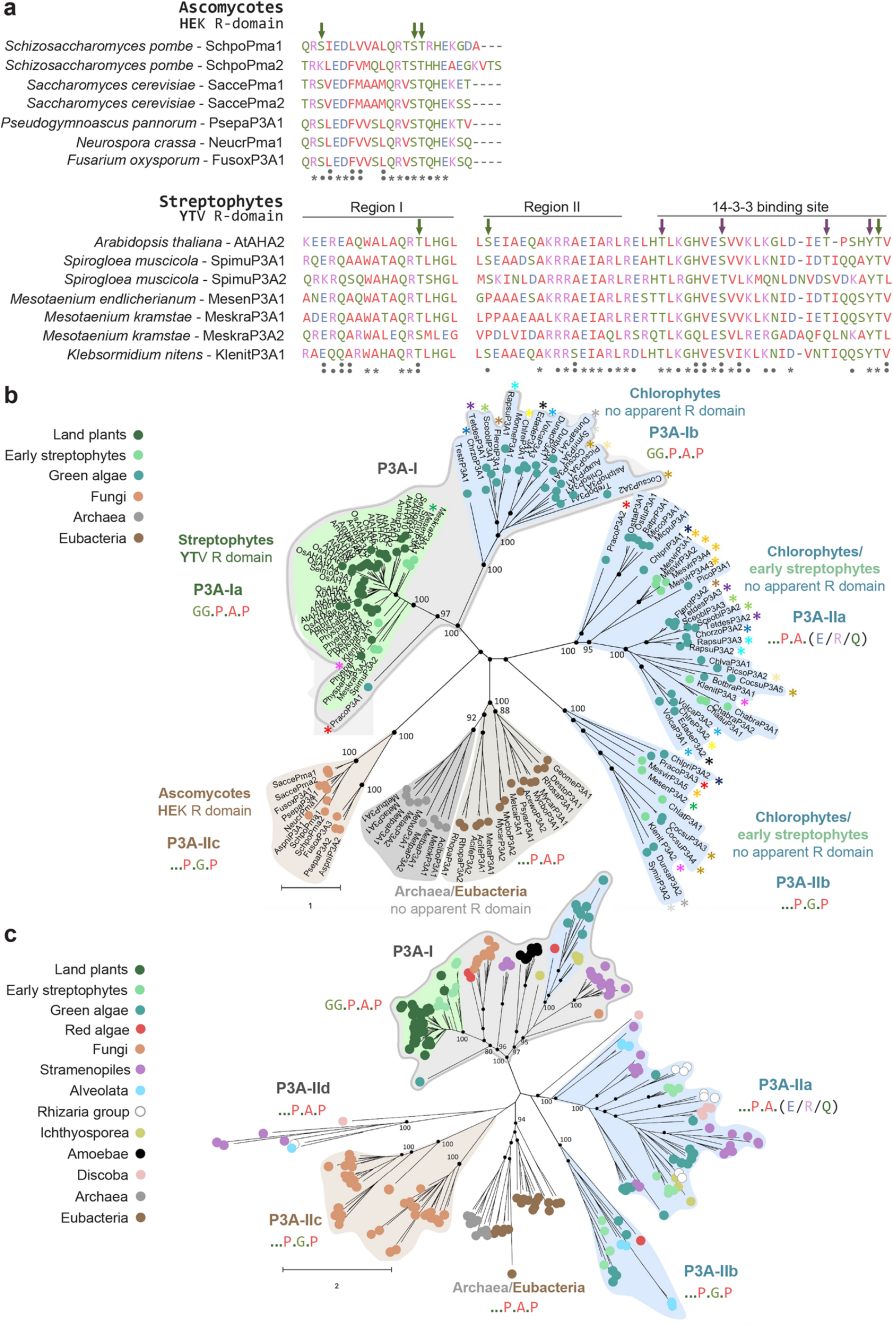
Sequences with a plant R domain are restricted to streptophytes. **a** Alignment of the C termini of plasma membrane H^+^-ATPase sequences from ascomycetes and streptophytes. Gray stars indicate conserved residues, two dots show high similarity, and one dot specifies similarity between the sequences. Autoinhibitory regions (Regions I and II) as well as the sequence that makes contact with 14-3-3 protein are indicated by lines for the Streptophytes. In vivo phosphosites are highlighted with arrows: green—phosphorylation activates the pump; purple—phosphorylation blocks activation. **b** Phylogenetic analysis of P3A plasma membrane H^+^-ATPase-like proteins from Viridiplantae reveal that sequences with a plant R domain are restricted to streptophytes. Sequences with a plant R domain (marked YTV; the T represents the Thr that has to be phosphorylated for 14-3-3 protein to bind) are only present in the streptophyte part of the P3A-I clade. The P3A-I clade is well-supported while the remaining eukaryotic clades are tentatively assigned as P3A-II subclades. The tree is the result of a maximum-likelihood analysis using RAxML and involving 141 amino acid sequences from 67 species as described in the section “Methods”. There was a total of 2112 positions in the final dataset. The best tree (likelihood−148 458.9268) after 1000 bootstrap rounds is shown. Colored asterisks indicate species with plasma membrane H^+^-ATPase-like sequences in more than one of the major clades. Abbreviated sequence names are given in full in Supplemental Table 1. Scale bar, 1 amino acid substitution per site. **c** Phylogenetic analysis of P3A plasma membrane H^+^-ATPase-like proteins from different domains of life reveal eukaryotic gene duplication events at the time before the evolution of present-day kingdoms. Sequences with a plant R domain are restricted to the streptophyte part, which includes early streptophytes and land plants, of the P3A-I clade. The P3A-I clade is well supported while the remaining eukaryotic clades are tentatively assigned as P3A-II subclades. The tree is the result of a maximum-likelihood analysis using RAxML and involving 247 amino acid sequences from 115 species as described in the section “Methods”. There was a total of 3 054 positions in the final dataset. The best tree (likelihood−270 139.98) after 1000 bootstrap rounds is shown. Scale bar, 2 amino acid substitutions per site. The trees were rooted with prokaryotic sequences (from Eubacteria and Archaea). In both cases, a separate Bayesian inference analysis was carried out using the program MrBayes, which resulted in similar trees. The Bayesian interference analysis was run for 1,000,000 generations and the average standard deviation of split frequencies between the resulting trees was 0.009507 for the tree in (**b**), and 0.018731 for the tree in (**c**). Black dots at nodes in the RAxML tree indicate maximum statistical support (*P* = 1) in the Bayesian inference analysis. Identified synapomorphies in transmembrane segment 4 (TM4) are given at the base of major clades. Each sequence in the tree is marked with a dot colored according to the taxonomic supergroup to which it belongs. Color codes are given in the figure.

To investigate the possible origin of the R domain of plant PM H^+^-ATPase, we used the complete hydrophilic carboxy-terminal sequence of 103 residues following the last (10th) transmembrane segment (TM10) of AHA2 as a bait to search the NCBI and the PhycoCosm Algal Genomics Resource databases. Hits were identified in all land plants (Embryophyta) investigated and, in addition, in a number of streptophyte algae, the closest relatives of land plants. In each of the predicted proteomes of four streptophyte algae, *Klebsormidium nitens* (Klebsormidiophyceae) and *Spirogloea muscicola*, *Mesotaenium kramstae* and *Mesotaenium endlicherianum* (all three from Zygnemophyceae) (Fig. 1), we identified a PM H^+^-ATPase with a perfectly conserved R domain (Figs. 1 and 2a) in addition to isoforms in *K. nitens* and *M. endlicherianum* without an R domain (Fig. 2b). The sequences include a threonine at the penultimate position, the phosphorylation of which is known to be required for 14-3-3 protein to bind, and other known phosphorylation sites conserved in all land plants from mosses to angiosperms including Thr881, Thr924, Ser931, Thr942, Tyr946, and Thr947^11,27^ (Fig. 2a). No structure with significant homology to the land plant R domain was observed in any predicted protein of green alga (Chlorophyta) and the early diverging Prasinodermophyta (Fig. 1) or in any other protein in the NCBI protein database when Streptophyta was excluded from the search.

The PM H^+^-ATPase (Pma1) of the ascomycete yeast *Saccharomyces cerevisiae* also has a well-characterized R domain^28^, albeit shorter (about 39 residues characterized by a C-terminal **HE**K sequence; conserved residues in bold face), and cryo-EM structures of this pump^15^ and the related *Neurospora crassa* pump^14^ demonstrate that it interacts with a neighboring pump organized in a hexamer. To investigate a possible relationship with plant plasma membrane H^+^-ATPases, the 39 last carboxy-terminal residues of *S. cerevisiae* were blasted against all predicted polypeptides in the NCBI database. Significant homology was found in all members of Ascomycota investigated (Fig. 2a, Supplemental Fig. 1) whereas no hits were retrieved outside Ascomycota besides the C-terminal domain of a zygomycete plasma membrane H^+^-ATPase (*Basidiobolus meristosporus*; ORX81303). This would suggest the R domains of streptophyte and ascomycete plasma membrane H^+^-ATPases each are unique entities that evolved independently.

To investigate the evolutionary relationship between PM H^+^-ATPases, we extracted predicted PM H^+^-ATPase sequences from genomes of representative species from green plants (Viridiplantae comprising Prasinodermophyta, Chlorophyta, and Streptophyta) and subjected them to phylogenetic analysis with ascomycete and prokaryote sequences as outgroups. The result was a phylogenetic tree with a prokaryotic clade and three Viridiplantae clades (Fig. 2b; Supplemental Table 1). Among the clades, one (designated P3A-I), which included all the sequences from land plants and sequences from most green algae, was characterized by the sequence synapomorphy **GG**IPI**A**M**P** in transmembrane segment 4 (TM4). Within the P3A-I clade, a few sequences of streptophyte algae together with all land plant PM H^+^-ATPase sequences formed a monophyletic clade, which did not include any sequence from green algae (Chlorophyta), and all sequences with a plant R domain belonged to this clade (Fig. 2b, c; Supplemental Fig. 2; Supplemental Table 1). The phylogenetic relationship between the remaining clades (designated P3A-IIa and P3A-IIb) was uncertain as statistical values at the diverging nodes were low. Ascomycetal PM H^+^-ATPases grouped in a monophyletic clade (here named P3A-IIc) with no affinity for the P3A-I clade provide further support that they are distantly related to land plant sequences.

In many species, more than one PM H^+^-ATPase was found to be present, and in some streptophyte algae and in most green algae members of both the P3A-I and P3A-II clades were represented in their genomes (marked with asterisks in Fig. 2b). Sequences of the streptophyte algae *Chara* and *Chloropkybus atmophyticus* were only represented in the P3A-II clades. This would suggest that the progenitor of Viridiplantae had both P3A-I and P3A-II ATPases but that P3A-II ATPases were lost during the evolution of Streptophyta towards land plants.

To investigate in a broader context the evolutionary relationship between PM H^+^-ATPases, we included in the phylogenetic analysis predicted PM H^+^-ATPase sequences from other major eukaryotic groups. The result was a phylogenetic tree with a prokaryotic clade and five major eukaryotic clades, four of which had already been assigned in the previous tree (Fig. 2c; Supplemental Fig. 2; Supplemental Table 1). The only new clade (designated P3A-IId) included sequences from the group of SAR (Stramenopiles, Alveolata, and Rhizaria). None of the major clades was unique for Viridiplantae. For example, in addition to sequences from all land plants and most green algae, Clade P3A-I now included sequences from such diverse groups as Stramenopiles, fungi, Ichthyosporea, and Amoebae. Several species of green algae, fungi and Stramenopiles had predicted PM H^+^-ATPases belonging to both the P3A-I and the P3A-II clades (Supplemental Table 1). This would suggest that the PM H^+^-ATPase underwent one or more gene duplications at a very early stage in the evolution of eukaryotes, and before the emergence of present-day kingdoms. Similar evidence of ancestral gene duplications has been observed before in other P-type ATPase subfamilies^29^.

### Generation of C-terminally truncated variants of AHA2

**Fig. 3:**
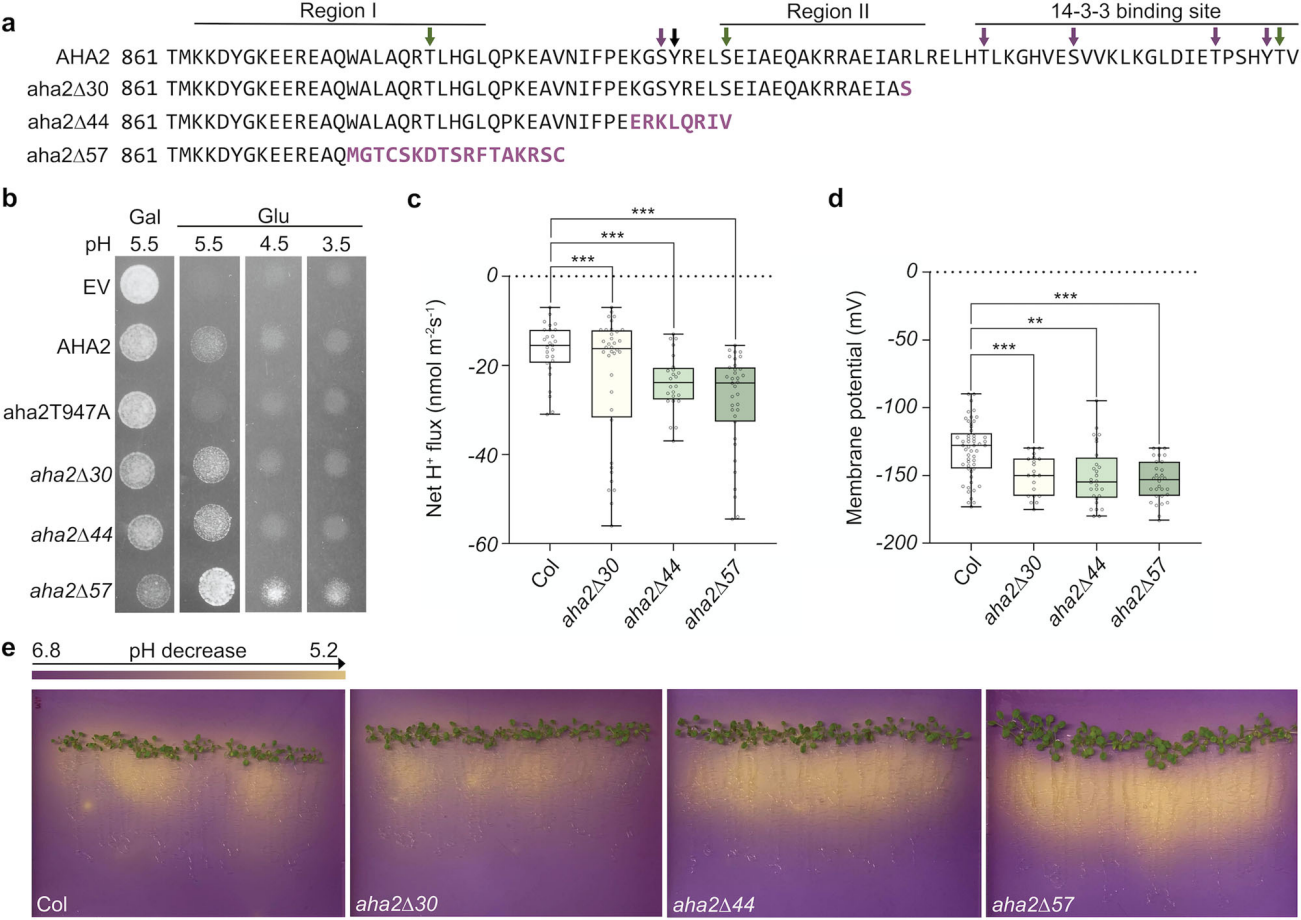
Truncation of the C-terminal autoinhibitory region of AHA2 increases pump activity in vivo. **a** Indels in the AHA2 mutant lines generated by CRISPR-Cas9 resulted in premature stop codons resulting in the truncation of the C-terminal autoinhibitory domain. *aha2Δ30*: line containing a 30-residue deletion with the predicted appearance of an additional nonsense residue. This truncation led to the removal of the 14‑3-3 protein binding site. *aha2Δ44*: line containing a 44-residue deletion with a predicted appearance of eight nonsense residues. This truncation also removes Region II. *aha2Δ57*: line containing a 57-residue deletion and predicted appearance of 17 nonsense residues. This truncation furthermore removes half of Region I, including a conserved phosphorylation site (Thr881) known to activate the protein independent of 14-3-3 protein. Nonsense amino acid residues are marked with purple bold lettering. Green arrows: phosphorylation activates AHA2; purple arrows: phosphorylation blocks activation of AHA2; black arrow: effect of phosphorylation is unknown. **b** Complementation of the yeast strain lacking endogenous plasma membrane H^+^-ATPase indicates that by truncating 57 amino acids from the C-terminal autoinhibitory domain, AHA2 is able to sustain yeast growth even when the pH of the medium drops to 3.5, suggesting a more active pumping of protons. aha2T947A functions as a negative control since the mutation leads to an inactive pump. The experiment was repeated three times with similar results. **c** Net H^+^ fluxes measured from the elongation root zones of 4–5 individual 6-day-old *A. thaliana* seedlings from each line with 3–9 measurements per seedling (*n* ≥ 24; *P* < 0.001; ***, pairwise *t*-test). **d** Truncation of the AHA2 C terminus leads to decreased membrane potential in 6-day-old *A. thaliana* roots with 3–9 measurements made in 3–8 individual plants from each line (*n* ≥ 21; **P < 0.01; ***P < 0.001; pairwise *t*-test). **e** Visualization of increased acidification capacity around the root system of approximately 2-week-old plants. To detect the medium’s pH change, bromocresol purple was applied at pH 6.5. The experiment was repeated three times with similar results. The boxes extend from the 25th to 75th percentiles, with whiskers ranging from minimum to maximum values. The centerlines indicate the medians and the black circles show each data point from the measurements.

To investigate the physiological importance of the R domain, we designed a CRISPR/Cas9-based strategy to sequentially remove in vivo the 14-3-3 protein binding site, Region II, and Region I, respectively, from the *A. thaliana* PM H^+^-ATPase *AHA2* (Fig. 3a). Sequencing of mutant plants showed that in all cases a single nucleotide (A or T) had been inserted into the wild-type (WT) *AHA2* DNA sequence, which resulted in frameshift mutations and, thus, in premature stop codons further downstream of the insertion site (Supplemental Fig. 3). In the *aha2Δ30* line, the insertion of an extra T gave rise to a codon encoding a Ser residue followed by the appearance of a premature stop codon, which resulted in the deletion of 30 C-terminal amino acid residues. In the case of *aha2Δ44*, the C terminus was truncated by 44 amino acids followed by 8 nonsense amino acids. *aha2Δ57* had the biggest deletion of 57 residues with an additional chain of 17 nonsense amino acid residues.

To test whether the additional nonsense residues resulted in pumps with unexpected properties, we generated the same mutations in *AHA2* cloned into a yeast expression plasmid and tested whether they would complement a mutation in the corresponding gene, *PMA1*, in *Saccharomyces cerevisiae*. In the applied yeast strain, the native *PMA1* promoter is replaced by a galactose-dependent one. In the transformed yeast, both *PMA1* and *AHA2* were expressed in a galactose medium, while only the gene encoding the plant PM H^+^-ATPase was expressed on glucose. A fraction of the produced AHA2 is phosphorylated in yeast at the penultimate threonine residue, which promotes interaction with yeast endogenous 14‑3‑3 protein and thereby activates the pump^30^. As a negative control, we, therefore, expressed the mutant protein aha2T947A, which behaves as the WT pump but cannot be phosphorylated at the 947th position. Production of aha2∆30 and aha2∆44 proteins partially complemented *pma1* in yeast grown on glucose media when pumping against an external pH of 5.5 but failed to do so when the medium was pH 4.5 and 3.5 (Fig. 3b). The produced aha2∆57 protein supported growth at all pH values tested (Fig. 3b). This demonstrates that the mutant proteins, despite having some additional C-terminal residues, are fully functional in the yeast system and behave as activated pump proteins, with aha2∆57 being the most active pump.

### Rhizosphere acidification and root growth of AHA2 mutants

To test directly whether H^+^ transport and membrane potential formation at the PM were affected in the mutated plants, we applied a non-invasive microelectrode ion flux estimation (MIFE) technique^31,32^. At the root tips, net H^+^ fluxes were positive (implying net H^+^ uptake), but fluxes gradually became negative (implying net H^+^ efflux) until the mature root zone (about 2 mm from the tip), from where fluxes remained approximately constant along the length of the root (Supplemental Fig. 4). The region 2–3 mm from the tip was therefore used for the MIFE experiments. Compared to the WT, significantly increased H^+^ fluxes were observed for all of the mutant lines (Fig. 3c), and their membrane potentials were about −25 mV more negative than those of WT plants (Fig. 3d).

To visualize whether the mutations affected the acidification of the medium around the roots, bromocresol purple was applied, which turns yellow below pH 5.2 and purple above pH 6.8. The ability to acidify the medium varied between individual plants of each genotype (Fig. 3e). However, taken together and compared to the WT, visible medium acidification was increased by the *aha2∆44* mutation and even more so by the *aha2∆57* mutation.

**Fig. 4:**
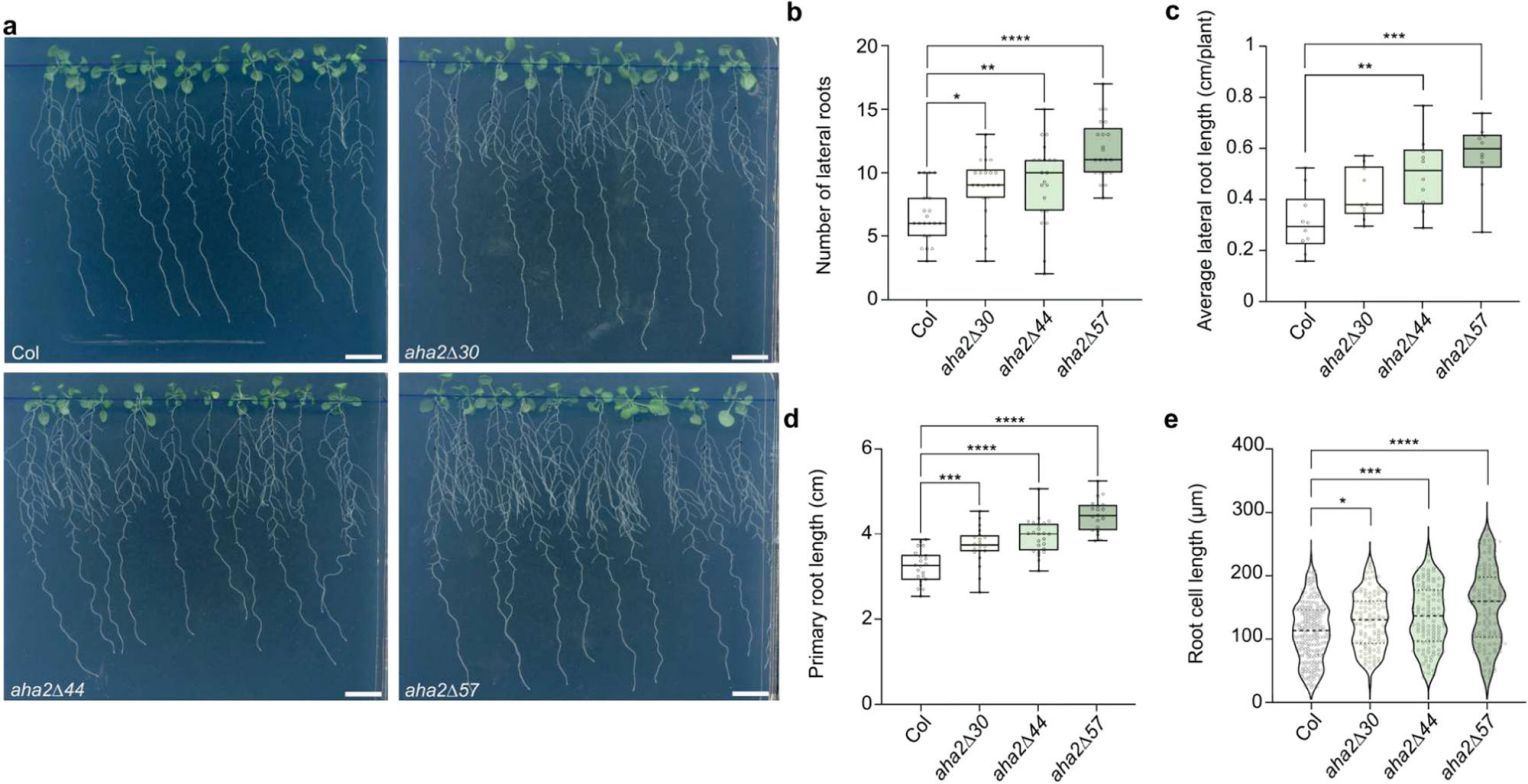
Truncation of AHA2 stimulates expansion growth in roots. **a** Representative photographs of the different *A. thaliana* mutant lines grown on ½ MS medium. The C‑terminal truncation of AHA2 results in longer side roots and a bushier root phenotype. Scale bar: 1 cm. **b** The mutant lines have increasingly more lateral roots per plant compared to the WT (*n* ≥ 20; *P  <  0.05; **P  <  0.01; ****P < 0.0001; one-way ANOVA with Tukey’s multiple comparison test). Similar results were obtained in three independent experiments. **c** The average lateral root length per plant is increased for the mutant plant lines (*n*  = 10; **P  <  0.01; ***P  <  0.001; one-way ANOVA with Tukey’s multiple comparison test). Similar results were obtained in three independent experiments. **d** The primary root length shows a slight gradual increase in correlation with the C-terminal truncation (*n* ≥ 19; ***P  <  0.001; ****P < 0.0001; one-way ANOVA with Tukey’s multiple comparison test). Similar results were obtained in three independent experiments. **e** The root cell length of five seedlings of each line was measured in the mature root zone. The analysis indicates a gradual and significant difference from the WT (*n* ≥ 110; *P < 0.05; ***P  < 0.001; ****P < 0.0001; one-way ANOVA with Tukey’s multiple comparison test). Similar results were obtained in three independent experiments. The boxes extend from the 25th to 75th percentiles, with whiskers ranging from minimum to maximum values. The centerlines indicate the medians and the black circles show each data point from these representative measurements. Violin plots show the probability density. The centerlines indicate the medians, the two other dashed lines the quartiles, and the black circles show each data point.

Increased medium acidification was associated with a bushy root phenotype, which was visible in the *aha2∆44* mutant but most pronounced in the *aha2Δ57* mutant (Fig. 4a). Increased number of lateral roots were observed (Fig. 4b), which were also longer than those of the WT (Fig. 4c), and the length of the primary root was increased (Fig. 4d). The increase in total root length corresponded with a significant increase in the length of individual cells in the mature root zone (Fig. 4e).

Gain-of-function *aha2* lines showed a significant reduction in primary root growth compared to the WT in response to high external concentrations of potassium, cesium, and arginine (Supplemental Fig. 5a–c) likely because they accumulated more of these compounds and reached a toxic level earlier. Furthermore, Perl’s blue staining of roots indicated that the *aha2Δ57* mutant accumulated more iron than the WT under control conditions (Supplemental Fig. 5d).

**Fig. 5:**
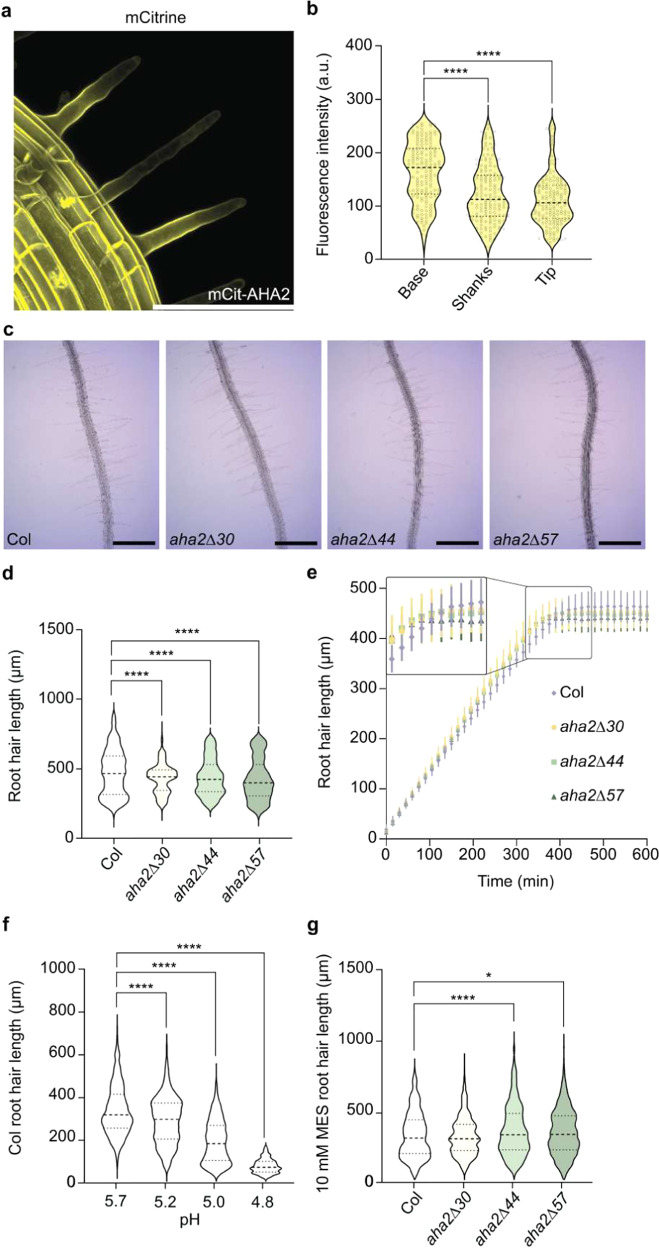
C-terminal truncation of AHA2 influences root hair growth. **a** Maximum intensity projection of AHA2 in mCitAHA2 root hairs. Yellow: mCitrine-AHA2. Scale bar: 100 µm. **b** Photons from mCitrine-AHA2 root hairs were counted. A significant difference was detected in the signal intensity at the base of the root hair compared to the shanks and the tip (*n* = 108; ****P  <  0.0001; one-way ANOVA with Tukey’s multiple comparison test). Violin plots show the probability density. The dashed centerlines indicate the medians and the two dotted lines the quartiles, and the black circles show each data point. **c** Representative images of WT and mutant plant root hairs grown for 3 days on ½ MS medium. Scale bar: 500 µm. **d** 3.5-day-old mutant root hairs are progressively shorter compared to the WT upon C-terminal truncation (*n* ≥ 874; ****P < 0.0001; one-way ANOVA with Tukey’s multiple comparison test). **e** The mutant root hairs first grow faster than those of the WT but after around 300 min the mutant root hairs stop growing while those of the WT continues to grow for an additional 100 min (*n* ≥ 9, error bars show SD). Inset: enlargement of the period during which the WT root hairs become longer than those of the mutants (boxed). **f** WT plants were grown for 2.5 days on ½ MS plates with different pH levels. As the pH decreased, the root hairs became progressively shorter (*n* ≥ 774; ****P  <  0.0001; one-way ANOVA with Tukey’s multiple comparison test). **g** When WT and mutant plants were grown for 2.5 days on a very well-buffered ½ MS medium with 10 mM MES, the difference shown in panel **d** was reversed and the mutant root hairs became longer than those of the WT (*n* ≥ 1036; *P  <  0.05; ****P  <  0.0001; one-way ANOVA with Tukey’s multiple comparison test).

For analysis of *AHA2* expression in root and root hairs, we used the mCitAHA2 line, which expresses the mCitrine-AHA2 translational fusion under the native promoter in *A. thaliana*^33^. We detected *AHA2* promoter activity in the roots and root hairs (Fig. 5a). Fluorescence of the reporter was strong at the PM of emerging root hairs and, as root hairs developed, fluorescence remained strong at the root hair base. However, fluorescence tended to generally decrease in the shanks of the root hair although it was never completely excluded from the tip (Fig. 5a, b).

Intriguingly, root hair growth showed an opposite phenotype as observed for the roots. Root hairs were progressively shorter in *aha2∆30*, *aha2∆44*, and *aha2Δ57* plants (Fig. 5c, d). When analyzing rates of root hair growth, we found that the initial growth of root hairs of *aha2∆30*, *aha2∆44*, and *aha2Δ57* was faster than that of the WT but slowed down after about 400 min, whereas WT root hairs continued to grow for 100 min more, which explained why they reached a longer length (Fig. 5e). An analysis of WT root hair growth on media with different acidities revealed that root hairs are shorter when acidity is lowered to pH 5.0 (Fig. 5f; Supplemental Fig. 6). Furthermore, the root hairs of the mutant plants were progressively longer than those of the WT (Fig. 5g) when plants were grown on a well-buffered ½ MS medium (10 mM MES). This observation demonstrates that it is the increased acidification of the medium and not the deregulated PM H^+^-ATPase per se that is the cause of the shorter root hairs observed in the mutant plants.

### C-terminal truncation of AHA2 results in smaller seed size

**Fig. 6:**
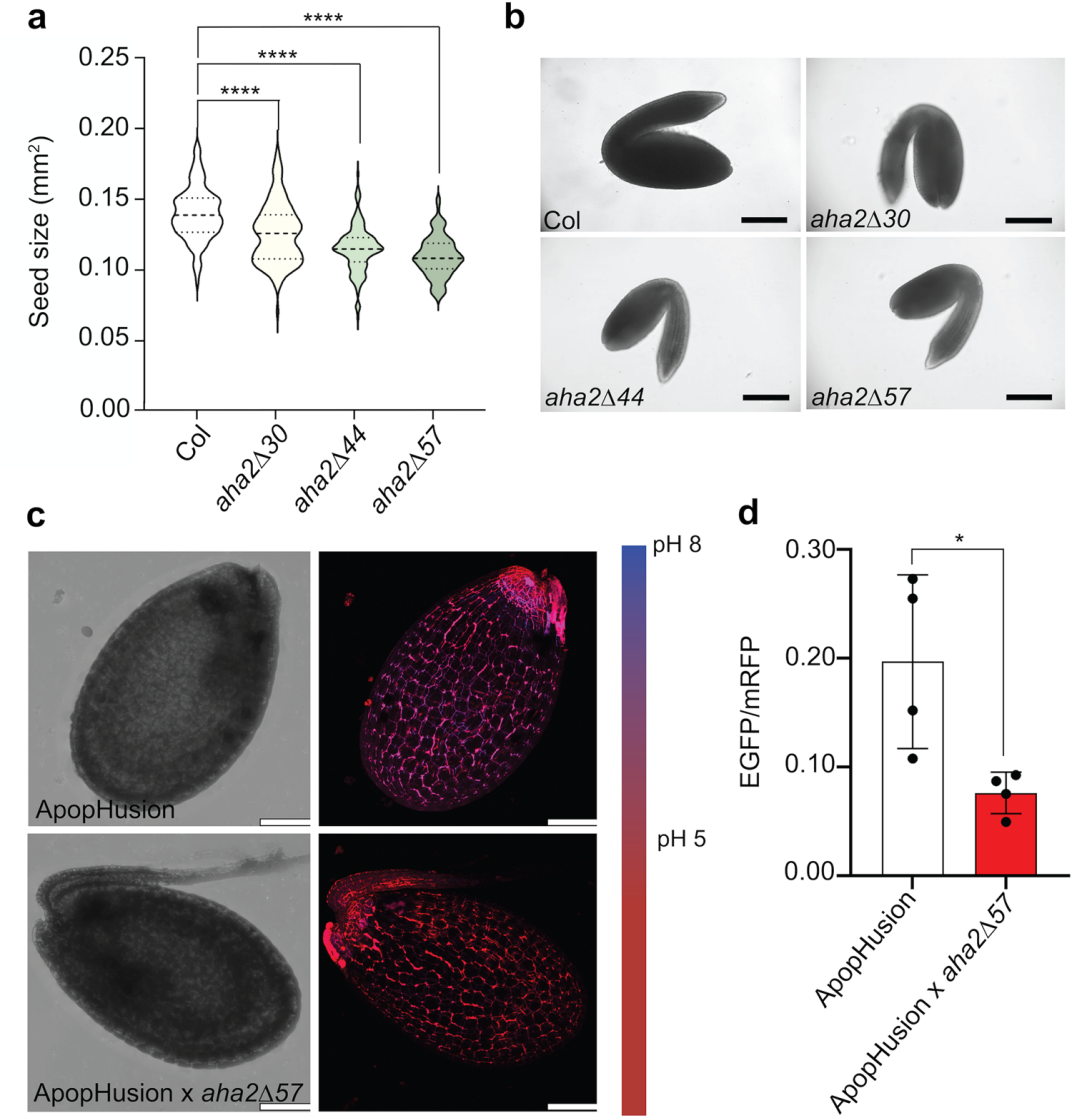
AHA2 C-terminal truncation results in smaller and acidified seeds. **a** The seed area of each line was analyzed using ImageJ (*n* = 130, ****P  <  0.0001; one-way ANOVA with Tukey’s multiple comparison test). Similar results were obtained from three biologically independent samples. Violin plots show the probability density. The dashed centerlines indicate the medians and the two dotted lines are the quartiles. **b** Representative images of WT and mutant embryos. Scale bar: 200 µm. **c** Maximum intensity projection overlay images of EGFP (blue) and mRFP1 (red) channels showing a representative signal of the genetically encoded pH sensor apopHusion in developing seeds. Scale bar: 100 µm. **d** The ratio between EGFP and mRFP1 was measured in four developing seeds of each line. The data points indicate the average value of the two fluorophore ratios in at least 107 randomly set regions of interest per seed (*n* = 4; *P  <  0.05; unpaired *t*-test, error bars show SD).

Seed size is an important parameter of yield in crops. It was therefore of interest to investigate whether activation of AHA2 had an impact on seed size. The size of seeds and embryos decreased progressively as the size of the C-terminal truncation of AHA2 increased (Fig. 6a, b). Despite the reduction in seed and embryo size, germination rates were identical in WT and mutant plants (Supplemental Fig. 7). When seeds were dissected and the fractions were subjected to ICP mass spectrometry, the relative distribution of potassium and other elements between the seed coat and embryo remained unchanged (Supplemental Fig. 8). This ruled out the possibility that activation of AHA2, and thus an increased membrane potential (negative on the inside), had interfered negatively with the export of potassium and other elements from the mother plant to the filial tissues.

**Fig. 7:**
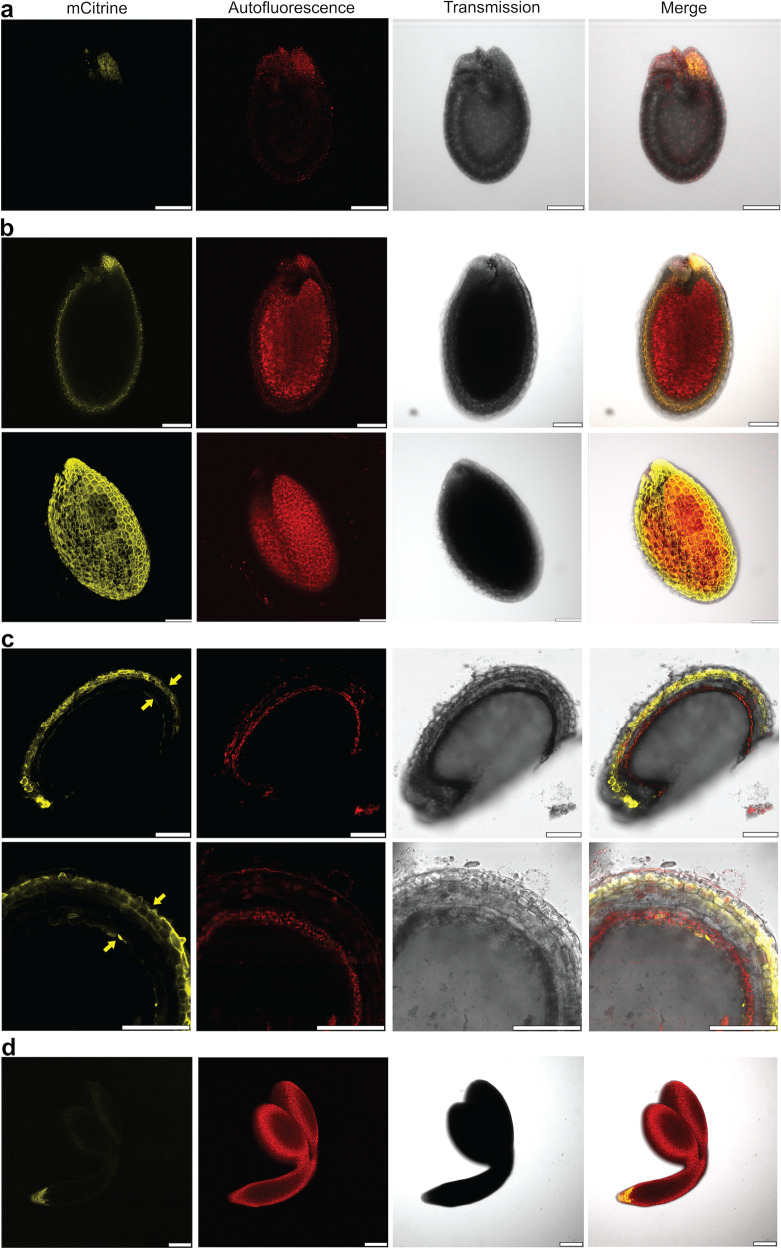
*AHA2* expression in developing seeds is confined to the micropylar endosperm region, the outer seed coat, and the endothelium. Confocal images of *AHA2* expression in developing mCitAHA2 seeds. Yellow: mCitrine-AHA2. Red: autofluorescence. Scale bar: 100 µm. **a** Maximum intensity projection of AHA2 in early-developing seeds. *AHA2* is expressed exclusively at the micropylar endosperm region. **b** Top panel: confocal image of an optical section of mature green seed. Bottom panel: maximum intensity projection of AHA2 distribution in mature green seed. The images show that at the mature stage AHA2 is highly expressed in the seed coat. **c** Confocal images of a mature green seed cut with a vibratome. Yellow arrows point to the endothelium and outer seed coat where *AHA2* is expressed. **d** Confocal images of an embryo at the mature green stage show that *AHA2* is expressed mostly in the radicle.

The *AHA2* promoter was active in seeds of mCitAHA2 transgenic plants (Fig. 7a–c), and we observed that, during seed development, *AHA2* expression follows a two-step path. Initially, expression was high in the micropylar endosperm region where phloem unloading to the radicle takes place (Fig. 7a), whereas at later stages, AHA2 was detected at different parts of the seed coat as well (Fig. 7b). *AHA2* expression was observed in the endothelium, the innermost cell layer of the seed coat facing the endosperm and developing embryo, and accumulated to higher levels in the outer seed coat layers (Fig. 7c). In the young embryo, the activity of the *AHA2* promoter was strong only in the tip of the radicle (Fig. 7d).

A possible explanation for the decreased seed size of the mutants could be that the embryos of mutant plants were subjected to an abnormal apoplastic pH. To investigate this possibility, we introduced the genetically encoded pH biosensor ApopHusion, which is exposed to the extracellular side of cells and permits live imaging of apoplastic pH, into the mutant lines. A comparison of the apoplastic pH in the developing seeds of ApopHusion and ApopHusion × *aha2Δ57* plants revealed that the pH of the seed apoplast was significantly lower in the mutant line (Fig. 6c, d). This observation raises the possibility that uncontrolled H^+^ efflux from the mother plant acidifies the filial tissues, resulting in smaller seeds.

### AHA2 mutants have wider stomatal apertures and are more susceptible to pathogen attack

**Fig. 8:**
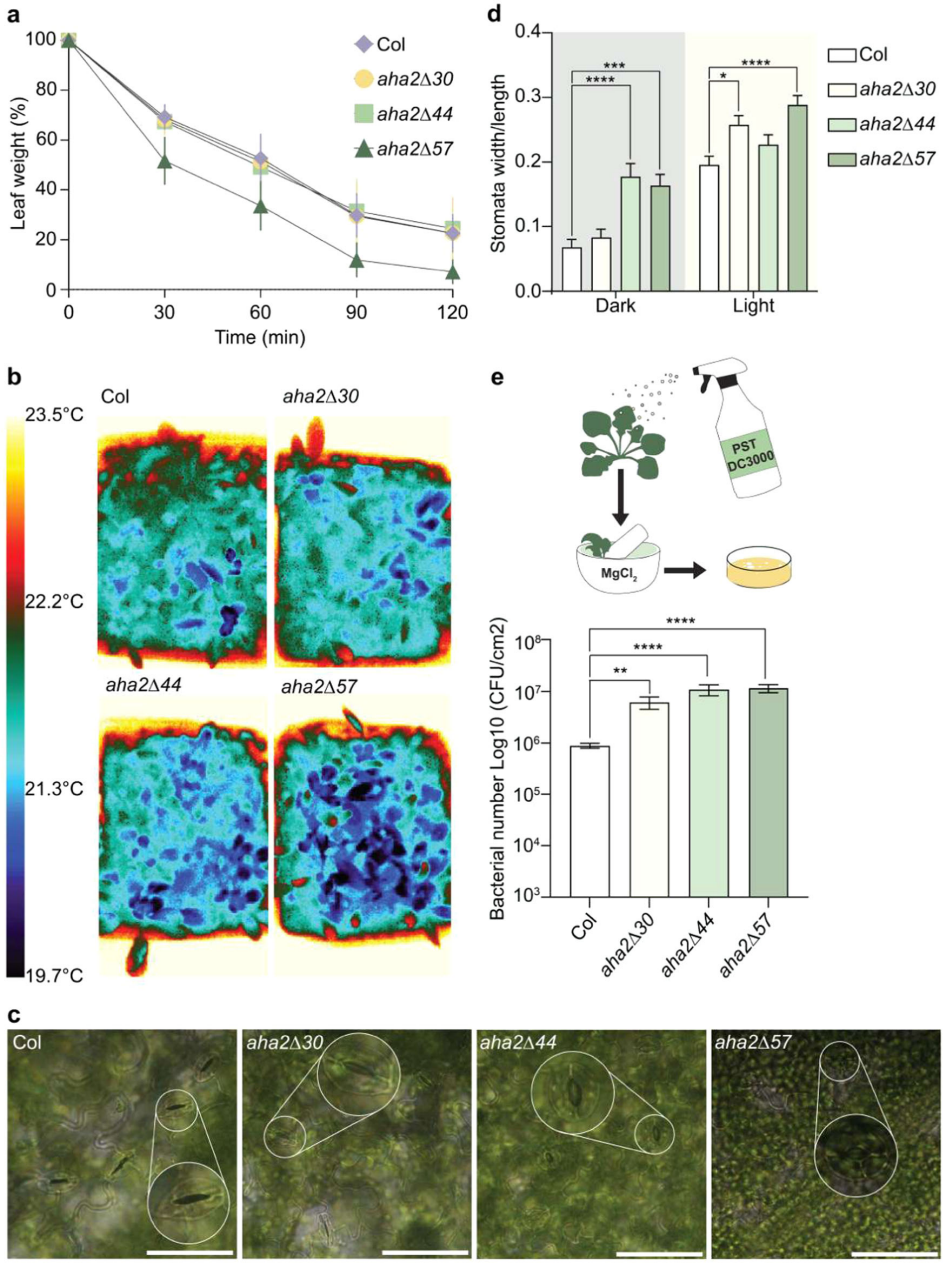
The AHA2 mutant plants have larger stomatal apertures and are more susceptible to water loss and pathogen attack. **a** The weight loss of detached leaves was monitored upon drying (*n* = 5, error bars show SD). Similar results were obtained in two independent experiments. **b** Infrared thermograph images of 12 three-week-old WT and mutant plants show increased water evaporation for the mutant lines possibly because of wider open stomata. Similar results were obtained in three independent experiments. **c** Representative images of leaf stomata after dark treatment. Scale bar: 50 µm. Larger white circle insets indicate enlargements of representative stomatal pores (smaller white circles). **d** Stomatal aperture size of dark- and light-treated leaves was measured with ImageJ (*n* ≥ 47; *P < 0.05; ***P < 0.001; ****P < 0.0001; two-way ANOVA with Tukey’s multiple comparison test, error bars show SEM). Similar results were obtained in two independent experiments. **e** Three-week-old plants were inoculated with *Pst*. Three days later, four times three leaf samples were harvested from each line for quantification of the bacterial populations (*n* = 4; **P < 0.01; ****P < 0.0001; one-way ANOVA with Tukey’s multiple comparison test, error bars show SD). Similar results were obtained in two independent experiments.

Stomatal guard cells control the opening and closing of the stomatal pores through which the plant takes up CO_2_ but also loses water as a result of transpiration. Even though AHA1 and AHA5 are the dominant PM H^+^-ATPases in guard cells, *AHA2* is also expressed at this location^34^. To test whether activation of AHA2 affects transpiration, we monitored the weight loss of detached leaves upon drying (Fig. 8a). Leaves of WT, *aha2∆30*, and *aha2∆44* mutant plants lost half of their weight in about 1 h, whereas a similar reduction in weight was seen already after 30 min for the *aha2∆57* mutant (Fig. 8a). Evaporation of water through stomatal pores leads to cooling of leaves^9^. Thermal imaging using an infrared camera revealed such an effect on the leaves of *aha2∆44* and *aha2∆57* plants, being most pronounced in *aha2∆57* plants (Fig. 8b). In the dark, stomatal pores of *aha2∆44* and *aha2Δ57* were already open to a degree, resembling stomatal pores of the WT in light (Fig. 8c, d). In the light, stomatal pores were open in both the WT and the mutants, but the apertures were significantly wider in the *aha2Δ57* mutant (Fig. 8d). Taken together, *aha2Δ44* and *aha2∆57* plants appear to have constitutively open stomatal pores, which lead to increased transpiration and water loss.

Pathogens invade leaf tissues through open stomatal pores^35^. To test whether activation of AHA2 affected the susceptibility of plants to pathogen attack, young plants were inoculated with *Pseudomonas syringae* pv. *tomato* DC3000 (Fig. 8e). After 3 days of incubation, colony-forming units were quantified. Compared to the WT, the degree of infection was significantly higher in the gain-of-function *aha2* mutant lines (Fig. 8e). This result illustrated that AHA2 activation increased the susceptibility of the plant to pathogen attack.

## Discussion

Here, we show that the complex R domain present in all plant PM H^+^-ATPases was already fully developed in some streptophyte algae with a tendency to terrestrialization but shows no homology to domains of PM H^+^-ATPases from other taxonomic groups including the R domain of ascomycete fungi (Figs. 1 and 2a). Convergent evolution of R domains in P-type ATPases is likewise evident in P2B PM Ca^2+^-ATPases of plants and animals that are controlled by autoinhibitory domains located in their N- and C-termini, respectively^36^. We further demonstrate that the R domain of the land plant *A. thaliana* PM H^+^-ATPase AHA2 controls cell elongation (Fig. 4), nutrient uptake, and growth (Supplemental Fig. 5). This provides evidence for the proposal that activation of the PM H^+^-ATPase increases solute uptake^1^ and supports the acid growth hypothesis^37,38^, according to which activation of PM H^+^-ATPase is a driver of elongation growth. This leads us to suggest that the R domain may have evolved to regulate growth and nutrient uptake already in the first land plants. In addition, loss of the R domain of AHA2 compromises the ability of *A. thaliana* to tolerate key abiotic and biotic stresses because stomatal pores, not present in streptophyte alga, are constitutively open, which results in water loss and provide access for invading microorganisms (Fig. 8).

AHA2 accumulates to high levels in roots and is also produced in root hairs. As the R domain of AHA2 was sequentially truncated, roots exported more protons to the extracellular medium (Fig. 3), elongation growth of root cells was stimulated, and roots grew longer, all in accordance with the acid growth theory^39,40^ (Fig. 4). The converse is also true as loss-of-function *aha2* mutants have shorter roots compared to the WT^41^. This supports the notion that apoplastic pH regulation via AHA2 is the main target for processes that control root growth^40^.

Gain-of-function mutant lines accumulated more Fe in their roots compared to the WT and showed increased sensitivity to elevated concentrations of monovalent cations in the growth medium (Supplemental Fig. 5). The converse was also reported by Haruta and Sussman (2012)^42^ for the *aha2* knockout line. Root hairs contribute directly to nutrient uptake^43–46^, and by providing the proton motive driving force for nutrient uptake, AHA2 has been proposed to contribute to this process^1^. When exposed to a well-buffered medium, root hairs expressing activated AHA2 grow longer (Fig. 5g) to compensate for the loss of rhizosphere acidification, which supports the assumption that AHA2 has a direct effect on root hair growth. In a less buffered control medium, gain-of-function mutants of AHA2 developed shorter root hairs (Fig. 5c, d) than the WT likely because an increased membrane potential and rhizosphere acidification facilitate nutrient uptake. It has previously been shown that low availability of nutrients leads to longer root hairs and the converse is also true^47–50^. In apparent support of the hypothesis that root hair length is negatively correlated with AHA2 activation, loss-of-function *aha2* mutants and an *aha2 aha7* double mutant have increased root hair lengths compared to the WT^41^, and application of a low concentration of vanadate (25 µM), which inhibits AHA2^51^, similarly causes an increase in root hair length^52^. Thus, we hypothesize that reduced root hair growth in response to AHA2 activation is likely an energy-saving developmental response to increased nutrient uptake.

Embryos of homozygous *aha1 aha2* double mutant embryos fail to develop, which is evidence that AHA1 and AHA2 are required for embryogenesis^53^. We found *AHA2* to be highly expressed at the micropylar endosperm domain close to the embryo radicle (Fig. 7a, b). The micropylar endosperm domain has been proposed to regulate embryo growth during early developmental stages by controlling the supply of hexose sugars^54,55^. AHA2 at this position could be involved in energizing the sugar loading of the embryo. However, an unexpected observation of this work was that seed size decreased progressively as the R domain was sequentially truncated (Fig. 6a). Decreased seed size was not accompanied by a decrease in germination rate (Supplemental Fig. 7), which suggests that embryo development was not impaired as much and that the differences in embryo size resulted from some form of growth inhibition during development. During the later stages of embryo development, AHA2 was detected also in the endothelium, the maternal cell layer that directly faces the filial tissues (Fig. 7c). Constitutive activation of AHA2 at this position causes increased apoplastic acidification (Fig. 6c, d). We hypothesize that the developing embryos of activated AHA2 mutants suffer from an unfavorable acidic external environment of the filial tissues, with growth inhibition as a likely result. Seed plants evolved much later than early land plants, which were spore-producing. It remains to be investigated how apoplastic pH affects spore development.

Stomatal pores of plants producing activated AHA2 were open even at night and showed increased rates of transpiration, which resulted in uncontrolled water loss (Fig. 8a–d). Furthermore, the transgenic plants were significantly more susceptible to attack by pathogens that invade internal tissues through these pores (Fig. 8e). An earlier study showed that overexpression of the *OSA1* PM H^+^-ATPase in rice resulted in increased plant growth, apparently without negative side effects, but in this case a pump with an intact R domain was studied^5^. The *ost2-1D* mutant of *A. thaliana* encodes a constitutively activated AHA1, a PM H^+^-ATPase related to AHA2 but predominantly expressed in shoots, and was shown to have wider stomatal apertures relative to the WT^9^.

Mosses have stomata in their sporophytic generation and the effect of PM H^+^-ATPase activation on stomatal pore size remains to be investigated. However, the liver moss *Marchantia polymorpha* encodes a PM H^+^-ATPase with a canonical plant R domain in which 14-3-3 protein binding is made possible by phosphorylation in response to light in a photosynthesis-dependent manner^27^. Regulation of stomatal pore opening likely predates the divergence of bryophytes and tracheophytes^56^ but, as the R domain of PM H^+^-ATPase was already fully developed in streptophyte algae, i.e. before the evolution of stomata, a likely first role of this domain was to control growth and nutrient uptake from dilute media such as slowly degrading rock surfaces.

It is interesting that Charophyceae lacks the R domain as it is present in the Klebsormidiophyceae that evolved earlier. However, as *Klebsormidium* can live in both water and soils^57,58^, in contrast to *Chara* which are exclusively aquatic algae, the lack in *Chara* of a PM H^+^-ATPase with an R domain may reflect gene loss. Chlorokyphoceae represented by its single species *Chlorokybus atmophyticus* live on soil and rock surfaces^59^ but branched out very early and before the appearance of the R domain, and thus might have adopted differently to terrestrial conditions. Zygnemophyceae are the closest relatives to land plants^60^ and PM H^+^-ATPases in this group have a fully developed R domain.

Taken together, these results establish the PM H^+^-ATPase AHA2 as a driver for acid growth and nutrient uptake in *A. thaliana*, but also demonstrate that tight regulation of this pump via the R domain is essential for fitness when this plant is exposed to environmental stresses that are unique to the terrestrial environment such as drought. As the R domain of *A. thaliana* had evolved to its present form already in streptophyte algae, the delicate post-translational regulation of PM H^+^-ATPase activity might have been required for the regulation of growth and nutrient uptake already in the first land plants.

## Methods

### Identification of P3A sequences

The strategy used to identify P-type PM H^+^-ATPase sequences was as previously reported^61^. Sequences were identified in the NCBI protein database using the Basic Local Alignment Search Tool (BLAST) program (http://blast.ncbi.nlm.nih.gov/) and used to search the genomes of 135 species representing major eukaryotic phyla and prokaryotes. For each species, BLAST searches were carried out using *A. thaliana* AHA2 (P-type P3A-I pump, P19456) or *S. cerevisiae* Pma1p (P-type P3A-II pump, P05030) amino acid sequences. Additional searches for P3A-type ATPase homologs were carried out through a BLAST search at the Joint Genome Institute (JGI) Genome Portal (http://genome.jgi.doe.gov/), the PlantGDB database (http://www.plantgdb.org/PpGDB/cgi-bin/blastGDB.pl#PPpep:Pp1s6_11V6.1), the PhycoCosm Algal Genomics Resource (https://phycocosm.jgi.doe.gov/phycocosm/home), and the Plantmorphogenesis server (http://www.plantmorphogenesis.bio.titech.ac.jp/~algae_genome_project/klebsormidium/klebsormidium_blast.html). All hits in each species with significant similarity to the query (expected value of <e−30) were selected and their relationship to the P3A-type ATPase subfamily was investigated by constructing phylogenetic trees for all candidate sequences in each individual genome together with a set of known P-type ATPases as described^29^. If sequences were included with maximal bootstrap support in the monophyletic P3A clade they were considered potential P3A ATPases. The nature of the individual sequences was subsequently confirmed by manual inspection for conserved sequence motifs characteristic of P-type P3A pumps^62^.

### Phylogenetic analysis of P3A ATPases

Protein sequence alignment was performed using MUSCLE^63^ implemented in MEGA6. This resulted in a total of 673 (177 amino acid sequences) and 1058 (111) amino acid residue positions in the final datasets of P3A pumps including all eukaryotic phylae (broad tree) and limited to Viridiplantae and Ascomycota (limited tree), respectively. The evolutionary history was inferred assuming an LG+INVGAMMA^64^ model, as identified by ProtTest^65^. Phylogenetic analyses were subsequently conducted using Bayesian inference and maximum-likelihood methods. Bayesian inference was performed with MrBayes 3.2.6^66^ and maximum-likelihood analyses with RAxML 8.2.9^67^ and, in initial analyses, MEGA6^68^. In the RAxML analyses, clade robustness was assessed with 1000 rapid bootstrap inferences followed by a thorough analysis of the maximum likelihood to obtain statistical support for the placement of nodes. The MrBayes analyses were performed using the following settings: eight chains of Markov chain Monte-Carlo iterations and a heated parameter of 0.05 with trees sampled every 1000 generations. The average standard deviations of split frequencies at termination of the analyses after 1,000,000 generations were 0.005606 for the broad P3A tree and 0.003138 for the limited P3A tree. Both the MrBayes and RAxML analyses were run on the CIPRES Science Gateway^69^ in the Extreme Science and Engineering Discovery Environment (XSEDE). Sequence synapomorphies specific to the clades identified were detected by manual inspection of protein sequences in each clade.

### Plant materials and growth conditions

The *Arabidopsis thaliana* seeds were surface sterilized by washing with 70% (v/v) ethanol for 15 min and then with 96% (v/v) ethanol for 20 min, followed by rinsing with sterile Milli-Q water. The growth media for the plants contained 2.165 g l^−1^ half-strength Murashige and Skoog salts, 1% (w/v) sucrose, 0.05% (w/v) MES (2-(*N*-morpholino) ethanesulfonic acid), and 0.8% (w/v) agar (pH 5.7). Seeds were stratified at 4 °C for 48 h to synchronize germination. In the growth chamber, the plants were subjected to 16-h-light/8-h-dark cycles (125 μmol photons m^−2^ s^−1^), and the temperature was a constant 20 °C.

Before the seeds were sowed in soil, they were stored in water at 4 °C in the dark to ensure stratification. In the growth chamber, the plants were subjected to 16-h-light/8-h-dark cycles (125 μmol photons m^−2^ s^−1^), and the temperature was a constant 20 °C.

The mCitrine-AHA2 and ApopHusion *A. thaliana* lines were previously described^33,70^. The C-terminally truncated activated variants of *A. thaliana* AHA2 transgenic lines were generated in this study.

### Generation of C-terminally truncated activated variants of AHA2 mutant plants

Plasmids were generated essentially as described previously^71^ with minor modifications. The *Aar*I-digested pKI1.1R vector was ligated with the appropriate sgRNA inserts (Supplemental Table 2). The constructed plasmids were introduced into *Agrobacterium tumefaciens* GV3101 by electroporation and transformed into *A. thaliana* ecotype Col-0 by the floral dip method^72^. The T_1_ generation seeds showing red fluorescence and T_2_ generation non-fluorescent seeds were selected under a stereomicroscope (Leica M205FA; excitation at 510–560 nm, and emission at 590–650 nm).

### Generation of the yeast constructs AHA2, ahaT947A, aha2∆30, aha2∆44, and aha2∆57

The constructs used to express WT *AHA2*, *ahaT947A*, *aha2∆30*, *aha2∆44*, and *aha2∆57* for yeast complementation were generated based on the plasmid YEp351. The constitutive yeast native PM H^+^-ATPase promoter *PMA1* was employed to regulate the expression of the genes. The target gene fragments were amplified by PCR using phusion high-fidelity DNA-polymerase (NEB). Both the PCR products and YEp351 were digested with *Xho*I and *Xba*I restriction enzymes (NEB) and then ligated by the T4 DNA ligase (NEB). The inserted genes were fully sequenced.

### Yeast complementation

The constructs were transformed into the RS-72 *Saccharomyces cerevisiae* strain (*Mat a; ade1‑100 his4-519 leu2-3 leu112 pPMA1-pGAL1*)^73^. In this strain, *PMA1* is replaced by the galactose-inducible promoter *GAL*1^74^. The yeast was grown in a galactose medium, and a glucose medium was used to activate the heterologous expression of *AHA2* and its variants. For drop tests, the diluted yeast cells were spotted on synthetic selective media with different pH values. Subsequently, the strains were grown at 30 °C for 3 days.

### H^+^ flux and membrane potential measurements

The MIFE technique was employed for measuring H^+^ fluxes and membrane potentials of growing roots as described^75^.

### Root acidification assay

Ten to twenty plants from each line were grown from seed on ½ MS medium for approximately 2 weeks and subsequently transferred to ½ MS plates with 0.006% (w/v) bromocresol purple. Photographs were taken when the biggest difference was seen between the lines (after 1–3 days). The experiment was repeated three times with similar results.

### Root growth assay

To quantify primary, and lateral root length and number, ½ MS plates with 7- to 15-day-old seedlings were scanned with an office scanner. The measurements were carried out using ImageJ software. The experiments were repeated three times with similar results.

For the nutrient uptake assays, 5- to 7-day-old seedlings grown under control conditions were transferred either to ½ MS plates as control or to ½ MS plates supplemented with 3 mM Arg, 120 mM KCl, or 2.7 mM CsCl. The ends of the primary roots were marked with dots and 7 days later the root length after the dot was measured using ImageJ software. The experiments were repeated two times with similar results.

### Root cell length measurements

The roots of 4- to 7-day-old seedlings were dipped into 10 μg/ml PI in H_2_O for 10 min and then rinsed in H_2_O. The root cell size was measured in the mature root zone, which was identified by the presence of root hairs. PI was excited at 488 nm, and the emitted light was collected between 597 and 655 nm. Images were processed using Leica LAS X confocal software. The experiments were repeated three times with similar results.

### Root hair measurements

For the root hair measurements, 2.5- to 3.5-day-old seedlings were transferred from plates to channel slide imaging chambers or regular microscopy slides, and root hair images were recorded using a Leica DMR HC microscope. The length of the root hairs was measured using ImageJ software. For data shown in Fig. 5c–e, the seedlings were grown on ½ MS media; for Fig. 5f, the media pH was lowered to the indicated values; and for Fig. 5g, the MES concentration of the media was increased to a final concentration of 10 mM.

### Perls staining

Four- to six-day-old *A. thaliana* seedlings grown under control conditions were first soaked in 90% acetone and kept on ice for 30 min. The seedlings were washed with MilliQ water two times. Subsequently, the seedlings were treated with a 1:1 solution of HCl and potassium ferrocyanide for 10–20 min. After washing the seedlings two times with MilliQ water, the plants were soaked in a clearing solution that contained chloral hydrate, glycerol, and MilliQ water in a ratio of 8:1:2. The roots were examined with a light microscope (Leica DMR HC).

### Seed size measurements using ImageJ

Seeds were harvested once the siliques had turned completely brown. Approximately 200 dry *A. thaliana* seeds from each line were distributed in an empty Petri dish. The scanned images were analyzed using ImageJ software.

### mCitrine analysis

Confocal imaging of mCitrine-AHA2 was performed using a Leica SP5-X microscope equipped with a white light laser, except for the images shown in Figs. 5a and  7c, which were obtained using a Leica Stellaris 8 microscope equipped also with a white light laser. To visualize mCitrine‑AHA2 protein, the fluorophore was excited at 514 nm, and the emission signal was detected between 525 and 620 nm. For Figs. 5a, b, 7a, b bottom panel, Z-stacks were obtained by imaging ~60–90 optical slices for a *z*-depth range of ~150–200 µm. For Fig. 5b, photons were counted along the plasma membrane of root hairs of ten 5-day-old mCit-AHA2 seedlings. For Fig. 7c, the seeds were embedded in 5% agarose and cut with a vibratome into 20-µm sections. Images were processed using Leica Confocal Software.

Whole seedlings shown in Supplemental Fig. 4b were imaged using a Leica M205FA fluorescence dissecting microscope with bright-field and long-pass fluorescence emission settings (excitation 500–520 nm and emission 540–580 nm). WT nontransgenic seedlings were also viewed under identical conditions to ensure that any fluorescence signals detected were not due to endogenous autofluorescence.

### ApopHusion analysis

A Leica Stellaris 8 confocal microscope was used for the apopHusion analysis. EGFP was excited at 488 nm using a white light laser and detected between 500 and 550 nm, and mRFP1 was excited at 558 nm and detected between 600 and 630 nm. For Fig. 6c, d, Z-stacks were obtained by imaging ~60 optical slices for a z‑depth range of ~105 µm. For Fig. 6d, the ratio between EGFP and mRFP1 photons was measured in at least 100 randomly set regions of interest per seed. WT nontransgenic seedlings were also viewed with identical conditions to ensure that any fluorescence signals detected were not due to endogenous autofluorescence.

### Elemental analysis of *A. thaliana* seeds

The method used for elemental analysis is essentially as described previously^76^ with a few modifications. For dissection, a few seeds were rinsed three times in ultrapure water and soaked on wet Cleanroom Wipes (Berkshire, Pro-Wipe® 750 9″ × 9″ PW750.0909.20) placed in an acid-washed glass Petri dish (soaked for 2 h in 1 M HCL, followed by soaking twice in ultrapure Milli-Q for 1 h). Softened seeds were dissected into seed coat and embryo fractions with super alloy electron microscopy tweezers under a stereomicroscope. The samples were digested in 50 µl 1:1 of 70% (v/v) HNO_3_ and 15% (v/v) H_2_O_2_ and subsequently diluted to 3000 µl before ICP-MS analysis. All samples were diluted 1:1 before analysis to have enough volume available for a standard autosampler. Initial analysis showed that all elements of interest were still present in concentrations far above the limit of quantification. Mg, K, Mn, Fe, Cu, and Zn were measured at *m*/*z* ratios of 24, 39, 55, 56, 63, and 66, respectively. An accuracy of ±10% was obtained for Mg, K, Mn, Fe, Cu, and Zn. Data were acquired and processed using the MassHunter 4.5 Chromatographic software package. For external calibration, a custom-made multi-element standard was used (P/N 4400-ICP-MSCS, CPI International). The standard is non-equimolar and corresponds to the ratio between elements typically found in plants. The digestion procedure and elemental analysis were validated using the certified reference material (CRM) NIST 1515 Apple Leaf (National Institute of Standard and Technology).

### Weight loss of detached leaves

Three-week-old plants were incubated in a growth chamber in the dark for 20 h. Subsequently, five leaves of similar developmental stages were detached and weighed immediately on an analytical scale (Sartorius CPA225D). The samples were placed 20 cm below a lamp bulb at room temperature. The weight of the leaves was monitored every 30 min for 2 h. Weight loss of the leaves was calculated as a percentage of the initial fresh weight. The experiment was repeated two times with similar results.

### Infrared camera experiments

Four-week-old *A. thaliana* plants were incubated in a growth chamber in the dark for 20 h. Photographs were taken with an infrared camera (FLIR A35 sc, FLIR Systems AB, Sweden) and analyzed using FLIR ResearchIR software. The experiment was repeated three times with similar results.

### Stomatal pore size measurement

Three-week-old *A. thaliana* plants were incubated in a growth chamber in the dark for 20 h. Three leaves were detached and cut in half longitudinally from each line. The samples were then placed in Petri dishes containing stomatal aperture solution (5 mM KCl, 10 mM MES, and 50 μM CaCl_2_, pH 6.5) and kept in the growth chamber for an additional 3 h under two different conditions. The first half of the leaf was kept in darkness, and the second half of the same leaf was placed under light. Both the dark- and light-treated samples were examined using light microscopy (Leica DMR HC), and photographs of representative samples were taken. The width and length of the stomatal pores were measured using ImageJ. The experiment was repeated twice with similar results.

### Disease test

Three-week-old *A. thaliana* plants were incubated in a growth chamber in the dark for 20 h. Cultures of virulent *Pseudomonas syringae* pv. *tomato* (*Pst*) DC3000 were grown at 28 °C to late log phase in LB media supplemented with rifampicin (25 µg ml^−1^). *Pst* was then harvested and resuspended in 10 mM MgCl_2_ and 0.01% (v/v) Silwet L-77 to OD_600_ 0.1 and then spray-inoculated onto plants until runoff and leaf surfaces were evenly wet. To maintain high humidity, the plants were covered with a translucent lid for 1 day. Bacterial populations within the leaves were determined at 3 days post inoculation (dpi). A total of four rosettes per line were investigated and three leaf discs were sampled from the same rosette using a 0.6-cm-diameter cork. The leaf samples were ground in 200 ml 10 mM MgCl_2_ using a pestle mounted on an electric drill while adding 800 ml of 10 mM MgCl_2_. A 6× serial dilution was performed. Twenty microliters of each of the six dilutions were plated on LB plates supplemented with appropriate antibiotics and incubated at 30 °C for 3 days. Then, the number of colonies was counted for calculating colony-forming units per square centimeter of leaf tissue. The experiment was repeated twice with similar results.

### Statistics and reproducibility

Statistical analysis was performed using Prism6 (GraphPad). One-way ANOVA using Tukey’s multiple comparison test was applied unless otherwise stated. Replication efforts confirmed the results presented.

### Reporting summary

Further information on research design is available in the Nature Portfolio Reporting Summary linked to this article.

## Supplementary information

Supplementary Information

Description of Additional Supplementary Data

Dataset 1

Reporting Summary

## Data Availability

All data supporting the findings of this study are included in this published article, its Supplementary Information, and the Supplementary Data file. Any other data and material will be made available upon reasonable request.
