# Supplementary material for: The evolution of plant proton pump regulation via the R domain may have facilitated plant terrestrialization
Source: Commun Biol. 2022 Nov 29;5:1312. doi: 10.1038/s42003-022-04291-y (PMC9708826; doi:10.1038/s42003-022-04291-y)
Supplement: Supplementary file 1 — Supplementary Information [file 42003_2022_4291_MOESM1_ESM.pdf]

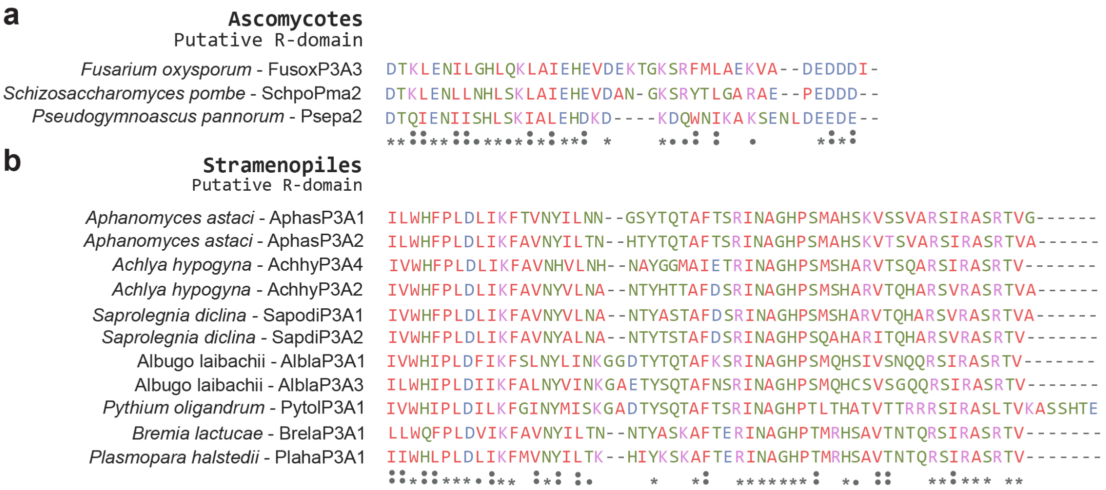

Supplemental Fig. 1: Alignment of C-termini PM H^{+}-ATPase sequences from Ascomycotes and Stramenopiles. Alignment of putative R domain sequences in **a,** Ascomycotes and **b,** Stramenopiles. Grey stars indicate conserved residues, two dots show high similarity, one dot specifies similarity between the sequences.

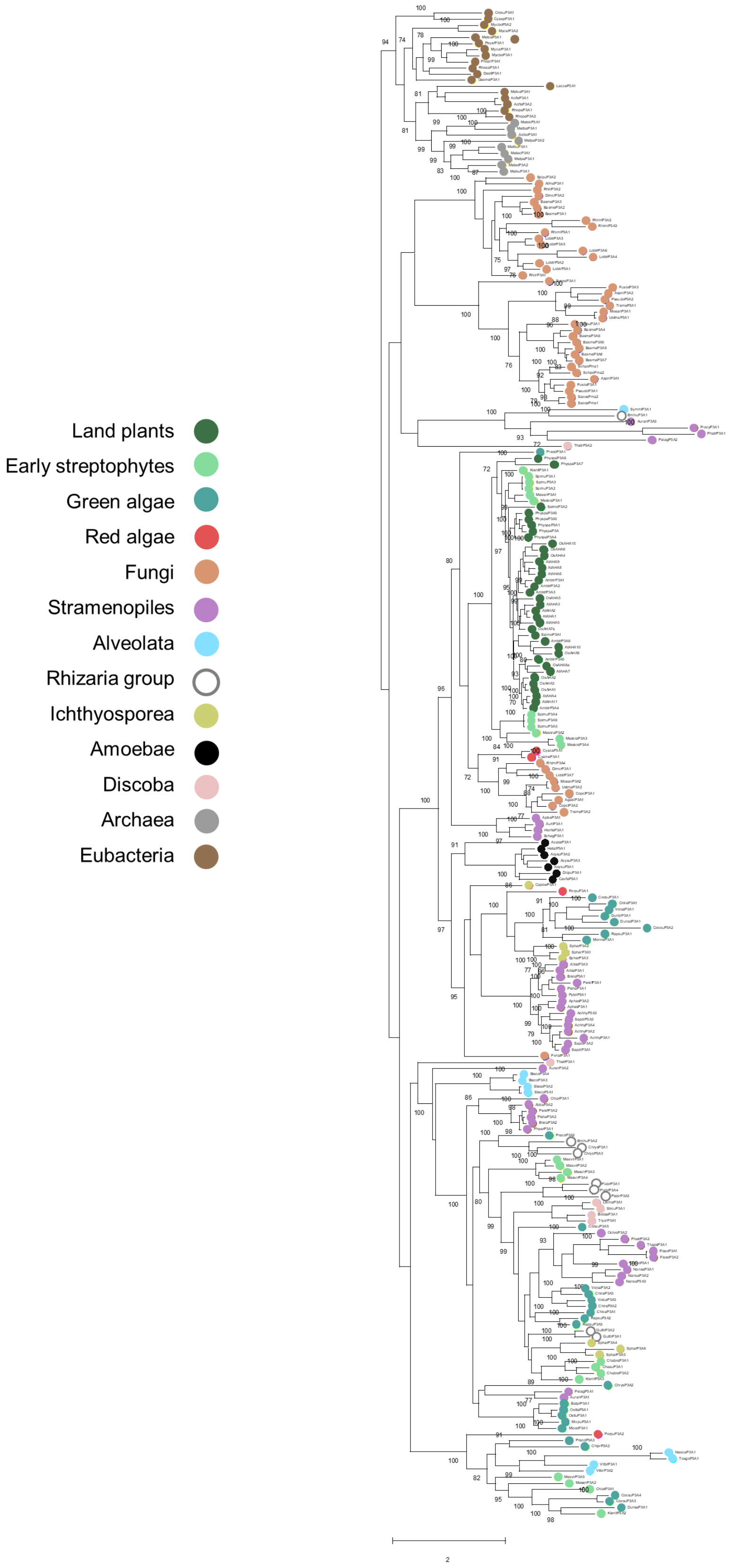

Supplemental Fig. 2: Phylogenetic analysis of P3A plasma membrane H^{+}-ATPase-like proteins from different domains. The tree is identical to that in Fig. 2 but here the abbreviated names of sequences are given at each branch.

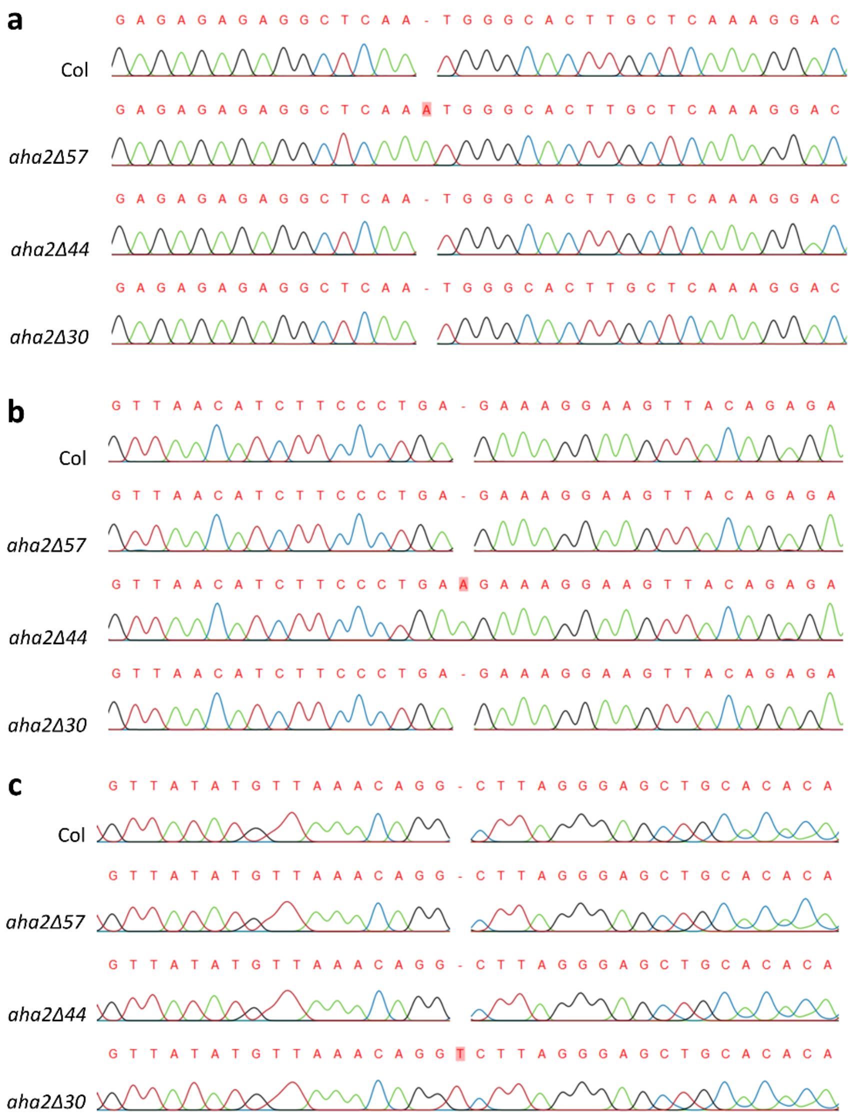

Supplemental Fig. 3. The CRISPR/Cas9-generated mutant lines with AHA2 C-terminal truncation. a-c, Sequencing data showing the indels introduced by CRISPR/Cas9 for the different mutant lines: **a,** *aha2Δ57*, **b,** *aha2Δ44*, and **c,** *aha2Δ30*. The shaded letters indicate the mutations.

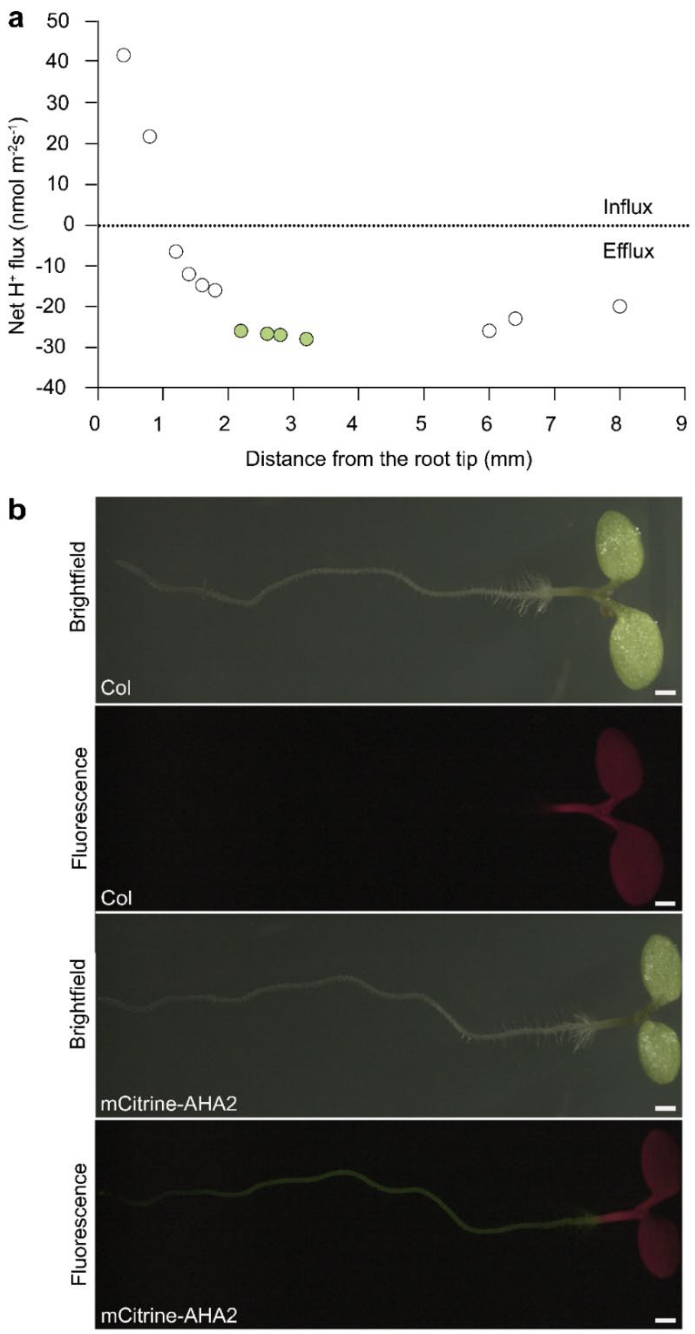

Supplemental Fig. 4: Net H^+ flux values and AHA2 expression level along the root axis. **a,** Net H^+ fluxes were measured from the root tip and upwards with the non-invasive microelectrode ion flux estimation (MIFE) technique. Green dots indicate the region used for the MIFE measurements. **b,** Fluorescence microscopy images of the accumulation of AHA2 fused to mCitrine in plant roots. Green: mCitrine, Red: autofluorescence. Scale bar: 0.5 mm.

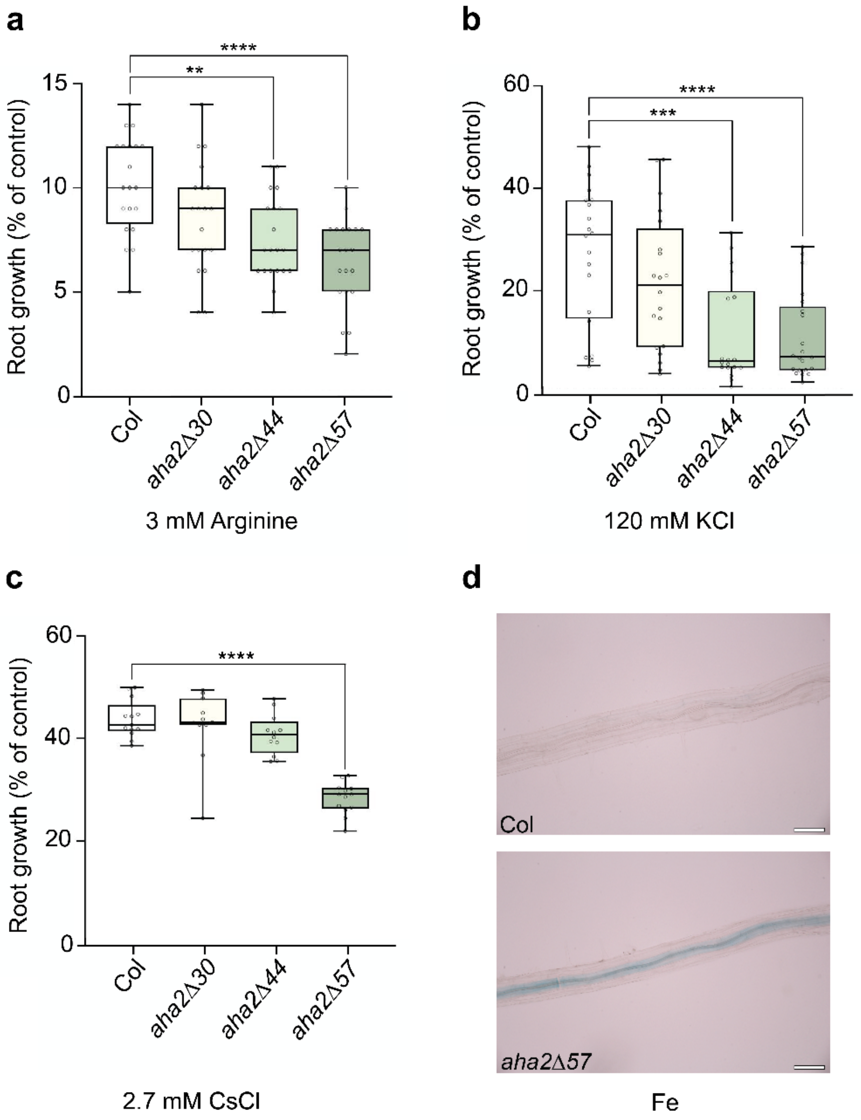

Supplemental Fig. 5: C-terminal truncation of AHA2 results in enhanced nutrient uptake. Growth responses of WT and gain-of-function AHA2 mutants to inhibitory external concentrations of **a,** arginine, **b,** potassium, and **c,** cesium. The mutant lines grew worse than the WT on the stress plates, indicating that they take up more nutrients and reach a toxic effect faster. The experiments were repeated two times with similar results. The values are percentages, where 100 is the control root growth on ½ MS media after transfer. The boxes extend from the 25th to 75th percentiles, with whiskers ranging from minimum to maximum values. The centerlines indicate the medians, and the black circles show each data point from the measurements. **a,** $n = 20$; **, $P < 0.01$; ****, $P < 0.0001$; ANOVA with Dunnett's multiple comparison test. **b,** $n \geq 18$; ***, $P < 0.001$; ****, $P < 0.0001$; ANOVA with Dunnett's multiple comparison test. **c,** $n \geq 11$; ****, $P < 0.0001$; ANOVA with Dunnett's multiple comparison test. **d,** Perls staining of WT and aha2Δ57 roots shows an increased iron uptake for the mutant line. Iron is indicated by the blue color. Scale bar: 100 µm.

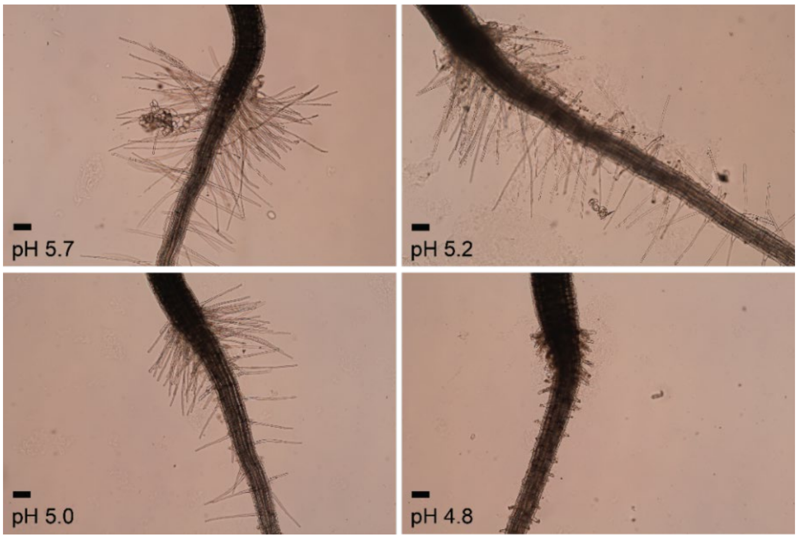

Supplemental Fig. 6: Lowering the pH of the growth medium inhibits root hair growth. Representative images of root hairs of WT seedlings grown for 3 days on ½ MS media with the indicated pH levels. Scale bar: 100 µm.

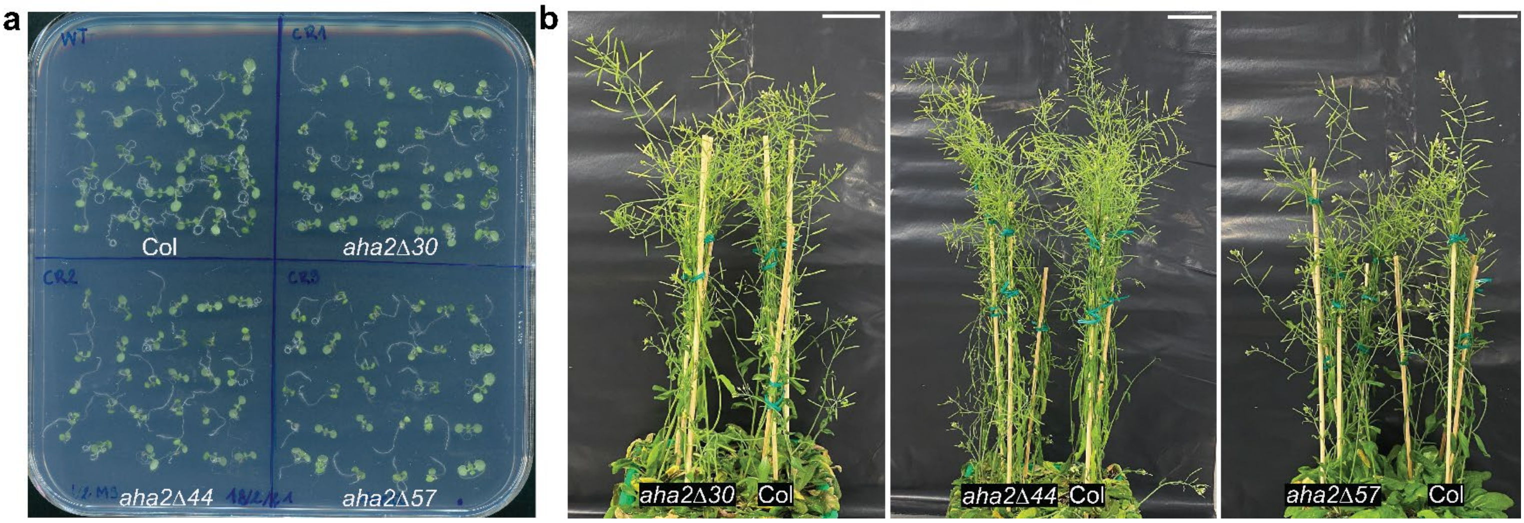

Supplemental Fig. 7: The WT and AHA2 truncation mutants have similar germination rates and full-size heights. a, Representative image of seed germination of the WT and AHA2 truncation mutants at 7 days after germination. **b,** Representative images of fully grown WT and mutant plants. Scale bar: 5 cm.

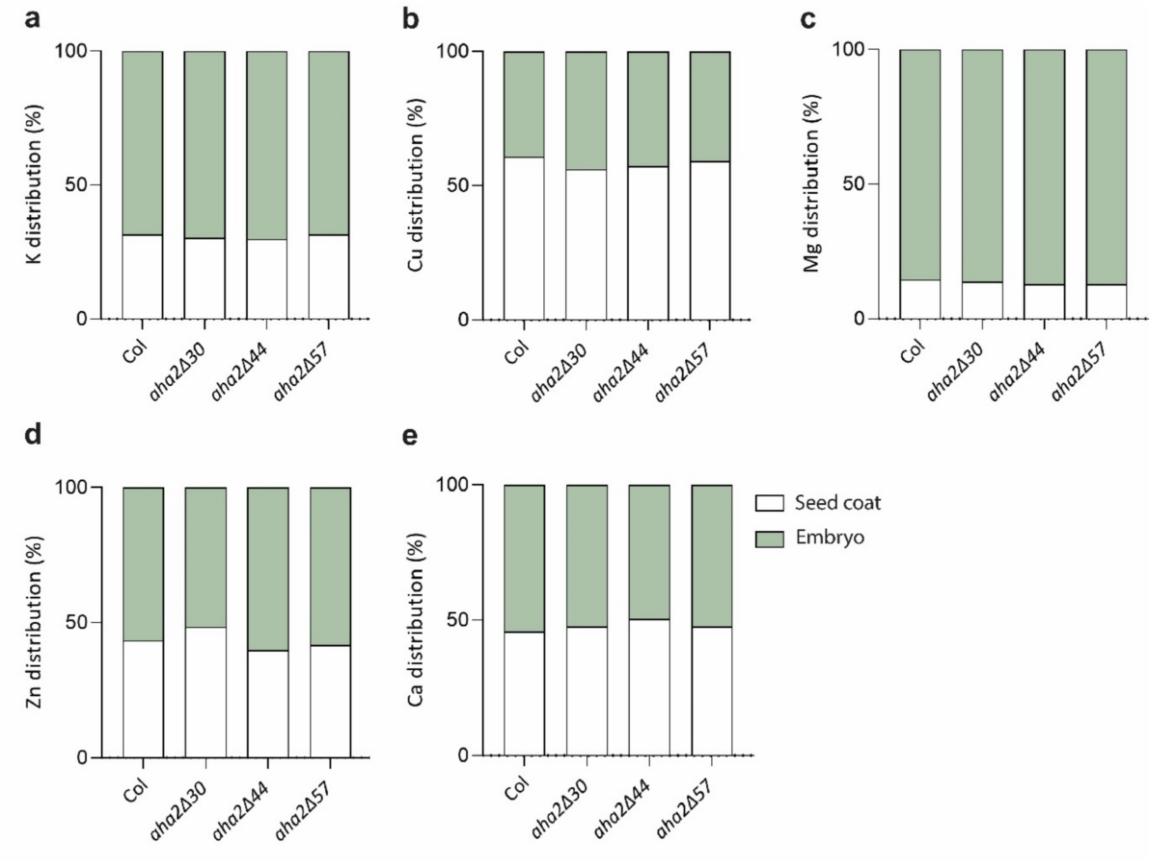

Supplemental Fig. 8: Relative distribution of elements between the seed coat and embryo does not differ between the WT and AHA truncation mutants. a-e, Microanalytical multi-elemental profiling of seeds separated into seed coats and embryos. The bars represent the total elemental content (100%). White represents the percentage values for the seed coat, while green shows the values for the embryos. No significant difference was observed in ANOVA with Dunnett's multiple comparison test. **a,** potassium **b,** copper **c,** magnesium **d,** zinc **e,** calcium.

Supplemental Table 1:

P3A plasma membrane H^+-ATPase-like proteins in selected organisms

<table>
<tr><th></th><th></th><th></th><th></th><th></th><th></th><th>Synapomorphies</th></tr>
<tr><th>Taxonomy</th><th>Species</th><th>Acc. Nr.</th><th>Abbrev. Name</th><th>Clade</th><th>R domain</th><th>TM4</th></tr>
<tr><td>Eukaryota</td><td></td><td></td><td></td><td></td><td></td><td></td></tr>
<tr><td>Bikonta</td><td></td><td></td><td></td><td></td><td></td><td></td></tr>
<tr><td>Rhodophyta</td><td></td><td></td><td></td><td></td><td></td><td></td></tr>
<tr><td>Bangiophyceae</td><td></td><td></td><td></td><td></td><td></td><td></td></tr>
<tr><td></td><td>Porphyridium purpureum</td><td>evm.model.contig_4410.18</td><td>PorpuP3A1</td><td>P3A-I</td><td>-</td><td>GGI PI AMP</td></tr>
<tr><td></td><td></td><td>evm.model.contig_3409.10</td><td>PorpuP3A2</td><td>P3A-IIb</td><td>-</td><td>AGAPVTLP</td></tr>
<tr><td></td><td>Galdieria phlegrea Soos</td><td>jgi|Galph1|4257|GphlegSOOS_G2885.1</td><td>GalphP3A1</td><td>P3A-I</td><td>-</td><td>GGI PI AMP</td></tr>
<tr><td></td><td>Cyanidium caldarium</td><td>BAA20486.1</td><td>CyacaP3A1</td><td>P3A-I</td><td>-</td><td>GGVPI AMP</td></tr>
<tr><td></td><td>Cyanidioschyzon merolae strain 10D</td><td>XP_005538169.1</td><td>CyameP3A1</td><td>P3A-I</td><td>-</td><td>GGVPI AMP</td></tr>
<tr><td>Viridiplantae</td><td></td><td></td><td></td><td></td><td></td><td></td></tr>
<tr><td>Prasinodermophyta</td><td></td><td></td><td></td><td></td><td></td><td></td></tr>
<tr><td></td><td>Prasinoderma coloniale CCMP1413</td><td>jgi|Praco1|16079|PRCOL_00005476-RA</td><td>PracoP3A1</td><td>P3A-I</td><td>-</td><td>GGVPI AMP</td></tr>
<tr><td></td><td></td><td>jgi|Praco1|16476|PRCOL_00005852-RA</td><td>PracoP3A2</td><td>P3A-IIa</td><td>-</td><td>ASI PI AMQ</td></tr>
<tr><td></td><td></td><td>jgi|Praco1|19933|PRCOL_00003206-RA</td><td>PracoP3A3</td><td>P3A-IIb</td><td>-</td><td>SVI PLGAP</td></tr>
<tr><td>Chlorophyta</td><td></td><td></td><td></td><td></td><td></td><td></td></tr>
<tr><td>Chlorodendrophyceae</td><td></td><td></td><td></td><td></td><td></td><td></td></tr>
<tr><td></td><td>Tetraselmis striata</td><td>jgi|Tetstr1|426842|TSEL_017057.t1</td><td>TetstrP3A1</td><td>P3A-I</td><td>-</td><td>GGI PI AMP</td></tr>
<tr><td>Chlorophyceae</td><td></td><td></td><td></td><td></td><td></td><td></td></tr>
<tr><td></td><td>Chlamydomonas reinhardtii</td><td>jgi|Chlre5_6|2361|Cre10.g459200.t1.2</td><td>ChlreP3A1</td><td>P3A-I</td><td>-</td><td>GGI PI AMP</td></tr>
<tr><td></td><td></td><td>jgi|Chlre5_6|11874|Cre03.g164600.t1.2</td><td>ChlreP3A2</td><td>P3A-IIa</td><td>-</td><td>ASI PI AI E</td></tr>
<tr><td></td><td>Volvox carteri</td><td>jgi|Volca2_1|12289|Vocar.0005s0158.1</td><td>VolcaP3A1</td><td>P3A-IIa</td><td>-</td><td>ASI PI AI E</td></tr>
<tr><td></td><td></td><td>jgi|Volca2_1|12290|Vocar.0005s0159.1</td><td>VolcaP3A2</td><td>P3A-IIa</td><td>-</td><td>ASI PI AI E</td></tr>
<tr><td></td><td></td><td>jgi|Volca2_1|6138|Vocar.0020s0178.1</td><td>VolcaP3A3</td><td>P3A-I</td><td>-</td><td>GGI PI AMP</td></tr>
<tr><td></td><td>Coccomyxa subellipsoidea C-169</td><td>jgi|Coc_C169_1|23431|estExt_Genewise1.C_70010</td><td>CocsuP3A1</td><td>P3A-I</td><td>-</td><td>GGI PI AMP</td></tr>
<tr><td></td><td></td><td>jgi|Coc_C169_1|49038|estExt_fgenesh1_pg.C_200136</td><td>CocsuP3A2</td><td>P3A-I</td><td>-</td><td>GGI PI AMP</td></tr>
<tr><td></td><td></td><td>jgi|Coc_C169_1|16699|e_gw1.10.11.1</td><td>CocsuP3A3</td><td>P3A-IIb</td><td>-</td><td>STVPVGMP</td></tr>
<tr><td></td><td></td><td>jgi|Coc_C169_1|16800|e_gw1.10.12.1</td><td>CocsuP3A4</td><td>P3A-IIb</td><td>-</td><td>SAMPVGMP</td></tr>
<tr><td></td><td></td><td>jgi|Coc_C169_1|66048|estExt_Genemark1.C_70348</td><td>CocsuP3A5</td><td>P3A-IIa</td><td>-</td><td>ASI PI AI E</td></tr>
<tr><td></td><td>Monoraphidium neglectum</td><td>XP_013899713.1</td><td>MonneP3A1</td><td>P3A-I</td><td>-</td><td>GGI PI AMP</td></tr>
<tr><td></td><td>Dunaliella bioculata</td><td>P54211.1</td><td>DunbiP3A1</td><td>P3A-I</td><td>-</td><td>GGI PI AMP</td></tr>
<tr><td></td><td>Dunaliella acidophila</td><td>P54210.1</td><td>DunacP3A1</td><td>P3A-I</td><td>-</td><td>GAI PI AMP</td></tr>
<tr><td></td><td>Dunaliella salina</td><td>jgi|Dunsal1_1|13784|Dusal.0554s00004.1</td><td>DunsaP3A1</td><td>P3A-I</td><td>-</td><td>GGI PI DTP</td></tr>
<tr><td></td><td></td><td>jgi|Dunsal1_1|13141|Dusal.0514s00005.1</td><td>DunsaP3A2</td><td>P3A-IIb</td><td>-</td><td>SVVPI GMP</td></tr>
<tr><td></td><td>Chromochloris zofingiensis SAG 211-14</td><td>jgi|Chrzof1|6285|Cz18g00060.t1</td><td>ChrzoP3A1</td><td>P3A-I</td><td>-</td><td>GGI PI AMP</td></tr>
<tr><td></td><td></td><td>jgi|Chrzof1|14728|Cz09g13190.t1</td><td>ChrzoP3A2</td><td>P3A-IIa</td><td>-</td><td>ASI PI AI E</td></tr>
<tr><td></td><td>Raphidocelis subcapitata</td><td>GBF93844.1</td><td>RapsuP3A1</td><td>P3A-I</td><td>-</td><td>GGI PI AMP</td></tr>
<tr><td></td><td></td><td>GBF93619.1</td><td>RapsuP3A2</td><td>P3A-IIa</td><td>-</td><td>ASI PI AI E</td></tr>
<tr><td></td><td></td><td>GBF97849.1</td><td>RapsuP3A3</td><td>P3A-IIa</td><td>-</td><td>ASI PI AI E</td></tr>
<tr><td></td><td>Tetradesmus deserticola SNI-2</td><td>jgi|TetrdesSNI2_1|7761814|fgenesh1_kg.19_#_11446_#_HAGSH_transcript/11606</td><td>TetdesP3A1</td><td>P3A-I</td><td>-</td><td>GGI PI AMP</td></tr>
<tr><td></td><td></td><td>jgi|TetrdesSNI2_1|7489503|fgenesh1_kg.6_#_26281_#_HAGSH_transcript/1418</td><td>TetdesP3A2</td><td>P3A-IIa</td><td>-</td><td>ASVPI AI E</td></tr>
<tr><td></td><td></td><td>jgi|TetrdesSNI2_1|7626405|fgenesh1_kg.12_#_11164_#_HAGSH_transcript/12177</td><td>TetdesP3A3</td><td>P3A-IIa</td><td>-</td><td>VSI PI AI E</td></tr>
<tr><td></td><td>Scenedesmus obliquus UTEX 393</td><td>jgi|Sceobl393_2|315672|CE315671_7051</td><td>SceoblP3A1</td><td>P3A-I</td><td>-</td><td>GGI PI AMP</td></tr>
<tr><td></td><td></td><td>jgi|Sceobl393_2|1958975|MIX6090_29_32</td><td>SceoblP3A2</td><td>P3A-IIa</td><td>-</td><td>ASVPI AI E</td></tr>
<tr><td></td><td></td><td>jgi|Sceobl393_2|1729856|fgenesh1_kg.2_#_4775_#_TRINITY_DN1527_c32_g1_i1</td><td>SceoblP3A3</td><td>P3A-IIa</td><td>-</td><td>VSI PI AI E</td></tr>
<tr><td></td><td>Flechtneria rotunda SEV3-VF49</td><td>jgi|Flerot1_1|11564099|fgenesh1_kg.18_#_32332_#_GZZNU_transcript/7538</td><td>FlerotP3A1</td><td>P3A-I</td><td>-</td><td>GGI PI AMP</td></tr>
<tr><td></td><td></td><td>jgi|Flerot1_1|11052115|fgenesh1_kg.4_#_32136_#_GZZNT_transcript/41587</td><td>FlerotP3A2</td><td>P3A-IIa</td><td>-</td><td>VSI PI AI E</td></tr>
<tr><td></td><td>Edaphochlamys debaryana CCAP 11/70</td><td>jgi|Edade1|5531|g4323.t1</td><td>EdadeP3A1</td><td>P3A-I</td><td>-</td><td>GGI PI AMP</td></tr>
<tr><td></td><td></td><td>jgi|Edade1|17155|g14688.t2</td><td>EdadeP3A2</td><td>P3A-IIa</td><td>-</td><td>ASI PI AI E</td></tr>
<tr><td>Trebouxiophyceae</td><td></td><td></td><td></td><td></td><td></td><td></td></tr>
<tr><td></td><td>Chlorella variabilis</td><td>XP_005843347.1</td><td>ChlvaP3A1</td><td>P3A-IIa</td><td>-</td><td>ASI PI AI E</td></tr>
<tr><td></td><td>Chlorella sorokiniana UTEX 1602</td><td>jgi|Chloso1602_1|8690|rna-gnl|WGS:LHPG|mrna.C2E21_3715A_C2E21_3715</td><td>ChlsoP3A1</td><td>P3A-I</td><td>-</td><td>GGI PI AMP</td></tr>
<tr><td></td><td>Botryococcus braunii Showa</td><td>jgi|Botrbrau1|4949|Bobra.0122s0027.1</td><td>BotbraP3A1</td><td>P3A-IIa</td><td>-</td><td>ASI PI AI E</td></tr>
<tr><td></td><td>Symbiochloris reticulata Spain</td><td>jgi|Dicre1|812443|fgenesh1_kg.703_#_2_#_Locus7252v1rpkm22.28</td><td>SymriP3A1</td><td>P3A-I</td><td>-</td><td>GGI PI AMP</td></tr>
<tr><td></td><td></td><td>jgi|Dicre1|838617|gm1.1896_g</td><td>SymriP3A2</td><td>P3A-IIb</td><td>-</td><td>CAI PVGMP</td></tr>
<tr><td></td><td>Auxenochlorella protothecoides UTEX 25</td><td>jgi|Auxpr25_1|2531|APUTEX2500001620-RA</td><td>AuxprP3A1</td><td>P3A-I</td><td>-</td><td>GGI PI AMP</td></tr>
<tr><td></td><td>Asterochloris glomerata Cgr/DA1pho</td><td>jgi|Astpho2|6147|Aster-08359</td><td>AstphoP3A1</td><td>P3A-I</td><td>-</td><td>GGI PI AMP</td></tr>
<tr><td></td><td>Picochlorum soloecismus DOE101</td><td>jgi|Picsp_1|520|NSC_00517-R2_plasma</td><td>PicsoP3A1</td><td>P3A-I</td><td>-</td><td>GGI PI AMP</td></tr>
<tr><td></td><td></td><td>jgi|Picsp_1|1918|NSC_05384-R1_p-type</td><td>PicsoP3A2</td><td>P3A-IIa</td><td>-</td><td>ASI PI AI E</td></tr>
<tr><td></td><td>Trebouxia sp. A1-2</td><td>jgi|TrebA12_1|9499|rna-gnl|WGS:VXIU|FRX49_10546-mRNA-1_mrna_FRX49_10546</td><td>TreboP3A1</td><td>P3A-I</td><td>-</td><td>GGI PI AMP</td></tr>
<tr><td>Mamiellophyceae</td><td></td><td></td><td></td><td></td><td></td><td></td></tr>
<tr><td></td><td>Micromonas pusilla CCMP1545</td><td>jgi|MicpuC3v2|7800|wlab.152942.1</td><td>MicpuP3A1</td><td>P3A-IIa</td><td>-</td><td>ASI PI ALR</td></tr>
<tr><td></td><td>Micromonas commoda NOUM17 (RCC 299)</td><td>jgi|MicpuN3v2|3205|wlab.234196.1</td><td>MiccoP3A1</td><td>P3A-IIa</td><td>-</td><td>ASI PI ALR</td></tr>
<tr><td></td><td>Ostreococcus tauri RCC4221</td><td>jgi|Ostta4221_3|74981|OT_ostta06g01380T0</td><td>OsttaP3A1</td><td>P3A-IIa</td><td>-</td><td>ASI PI ALR</td></tr>
<tr><td></td><td>Ostreococcus lucimarinus CCE9901</td><td>jgi|Ost9901_3|41354|e_gwEuk.6.668.1</td><td>OstluP3A1</td><td>P3A-IIa</td><td>-</td><td>ASI PI ALR</td></tr>
<tr><td></td><td>Bathycoccus prasinos</td><td>XP_007513114.1</td><td>BatprP3A1</td><td>P3A-IIa</td><td>-</td><td>ASI PI ALR</td></tr>
<tr><td>Chloropicophyceae</td><td></td><td></td><td></td><td></td><td></td><td></td></tr>
<tr><td></td><td>Chloropicon primus CCMP1205</td><td>jgi|Chlpri1|1764|rna-gnl|IITBIO|A3770_02p17640_mRNA_A3770_02p17640</td><td>ChlpriP3A1</td><td>P3A-IIa</td><td>-</td><td>ASI PI AMQ</td></tr>
<tr><td></td><td></td><td>jgi|Chlpri1|6456|rna-gnl|IITBIO|A3770_11p64560_mRNA_A3770_11p64560</td><td>ChlpriP3A2</td><td>P3A-IIb</td><td>-</td><td>ATLPVAMP</td></tr>
<tr><td>Picocystophyceae</td><td></td><td></td><td></td><td></td><td></td><td></td></tr>
<tr><td></td><td>Picocystis sp. ML</td><td>jgi|Pico_ML_1|52514|g386.t1</td><td>PicoP3A1</td><td>P3A-IIa</td><td>-</td><td>ASI PMAME</td></tr>
<tr><td>Streptophyta</td><td></td><td></td><td></td><td></td><td></td><td></td></tr>
<tr><td>Chlorokybophyceae</td><td></td><td></td><td></td><td></td><td></td><td></td></tr>
<tr><td></td><td>Chlorokybus atmophyticus CCAC 0220</td><td>jgi|Chlat1|2463|Chrsp171S02344</td><td>ChlatP3A1</td><td>P3A-IIb</td><td>-</td><td>SVVPI GMP</td></tr>
<tr><td>Mesostigmatophyceae</td><td></td><td></td><td></td><td></td><td></td><td></td></tr>
<tr><td></td><td>Mesostigma viride NIES-296</td><td>jgi|Mesvir1|14942|Mv05526-RA.1</td><td>MesvirP3A1</td><td>P3A-IIa</td><td>-</td><td>ASI PI AME</td></tr>
<tr><td></td><td></td><td>jgi|Mesvir1|22356|Mv17859-RA.1</td><td>MesvirP3A2</td><td>P3A-IIa</td><td>-</td><td>ASI PI AME</td></tr>
<tr><td></td><td></td><td>jgi|Mesvir1|11910|Mv00250-RA.1</td><td>MesvirP3A3</td><td>P3A-IIa</td><td>-</td><td>ASI PI AME</td></tr>
<tr><td></td><td></td><td>jgi|Mesvir1|2803|Mv13910-RA.1</td><td>MesvirP3A4</td><td>P3A-IIa</td><td>-</td><td>ASI PI AME</td></tr>
<tr><td></td><td></td><td>jgi|Mesvir1|8845|Mv02741-RA.1</td><td>MesvirP3A5</td><td>P3A-IIb</td><td>-</td><td>AVVPVGMP</td></tr>
<tr><td>Klebsormidiophyceae</td><td></td><td></td><td></td><td></td><td></td><td></td></tr>
<tr><td></td><td>Klebsormidium nitens NIES-228</td><td>jgi|Klenit1|1475|rna-KFL_000230090</td><td>KlenitP3A1</td><td>P3A-I</td><td>Plant-type</td><td>GGI PI AMP</td></tr>
<tr><td></td><td></td><td>jgi|Klenit1|11046|rna-KFL_004430040</td><td>KlenitP3A2</td><td>P3A-IIb</td><td>-</td><td>SVVPI GMP</td></tr>
<tr><td></td><td></td><td>jgi|Klenit1|4745|rna-KFL_001070030</td><td>KlenitP3A3</td><td>P3A-IIa</td><td>-</td><td>ASI PI AI E</td></tr>
<tr><td>Streptophytina</td><td></td><td></td><td></td><td></td><td></td><td></td></tr>
<tr><td>Charophyceae</td><td></td><td></td><td></td><td></td><td></td><td></td></tr>
<tr><td></td><td>Chara braunii S276</td><td>jgi|Chabra1|337449|rna-CBR_g30033-2_CBR_g30033</td><td>ChabraP3A1</td><td>P3A-IIa</td><td>-</td><td>ASI PLAI E</td></tr>
<tr><td></td><td></td><td>jgi|Chabra1|324545|CBR_g6273</td><td>ChabraP3A2</td><td>P3A-IIa</td><td>-</td><td>ASI PLAI E</td></tr>
<tr><td></td><td>Chara australis</td><td>AWC08675.1</td><td>ChaauP3A1</td><td>P3A-IIa</td><td>-</td><td>ASI PLAI E</td></tr>
<tr><td>Zygnemophyceae</td><td></td><td></td><td></td><td></td><td></td><td></td></tr>
<tr><td></td><td>Spirogloea muscicola CCAC 0214</td><td>jgi|Spimu1|21100|SM000057S18387</td><td>SpimuP3A1</td><td>P3A-I</td><td>+</td><td>GGI PI AMP</td></tr>
<tr><td></td><td></td><td>jgi|Spimu1|18312|SM000042S15336</td><td>SpimuP3A2</td><td>P3A-I</td><td>+</td><td>GGI PI AMP</td></tr>
<tr><td></td><td>Mesotaenium endlicherianum SAG 12.97</td><td>jgi|Mesen1|10597|ME000086S10133</td><td>MesenP3A1</td><td>P3A-I</td><td>+</td><td>GGI PI AMP</td></tr>
<tr><td></td><td></td><td>jgi|Mesen1|5913|ME000030S05179</td><td>MesenP3A2</td><td>P3A-IIb</td><td>-</td><td>AVVPI GMP</td></tr>
<tr><td></td><td>Mesotaenium kramstae Lemmermann NIES-657</td><td>jgi|Meskra657_3|2217935|estExt_Genewise1.C_1_t60016</td><td>MeskraP3A1</td><td>P3A-I</td><td>+</td><td>GGI PI AMP</td></tr>
<tr><td></td><td></td><td>jgi|Meskra657_3|3108489|estExt_fgenesh1_pm.C_90064</td><td>MeskraP3A2</td><td>P3A-I</td><td>+</td><td>GGI PI AMP</td></tr>
<tr><td>Embryophyta</td><td></td><td></td><td></td><td></td><td></td><td></td></tr>
<tr><td>Bryophyta (mosses)</td><td></td><td></td><td></td><td></td><td></td><td></td></tr>
<tr><td></td><td>Physcomitrella patens subsp. patens</td><td>XP_024366241</td><td>PhyspaP3A1</td><td>P3A-I</td><td>+</td><td>GGI PI AMP</td></tr>
<tr><td></td><td></td><td>XP_024392450</td><td>PhyspaP3A2</td><td>P3A-I</td><td>+</td><td>GGI PI AMP</td></tr>
<tr><td></td><td></td><td>XP_024371351</td><td>PhyspaP3A3</td><td>P3A-I</td><td>+</td><td>GGI PI AMP</td></tr>
<tr><td></td><td></td><td>XP_024362091</td><td>PhyspaP3A4</td><td>P3A-I</td><td>+</td><td>GGI PI AMP</td></tr>
<tr><td></td><td></td><td>XP_024371153</td><td>PhyspaP3A5</td><td>P3A-I</td><td>+</td><td>GGI PI AMP</td></tr>
<tr><td></td><td></td><td>XP_024384202</td><td>PhyspaP3A6</td><td>P3A-I</td><td>+</td><td>GGI PI AMP</td></tr>
<tr><td></td><td></td><td>XP_024390307</td><td>PhyspaP3A7</td><td>P3A-I</td><td>+</td><td>GGI PI AMP</td></tr>
<tr><td>Tracheophyta</td><td></td><td></td><td></td><td></td><td></td><td></td></tr>
<tr><td></td><td>Selaginella moellendorffii</td><td>jgi|Selmo1|430150|fgenesh2_pg.C_scaffold_125000029</td><td>SelmoP3A1</td><td>P3A-I</td><td>+</td><td>GGI PI AMP</td></tr>
<tr><td></td><td></td><td>jgi|Selmo1|76771|e_gw1.1.271.1</td><td>SelmoP3A2</td><td>P3A-I</td><td>+</td><td>GGI PI AMP</td></tr>
<tr><td></td><td>Amborella trichopoda</td><td>XP_020525755</td><td>AmbtrP3A1</td><td>P3A-I</td><td>+</td><td>GGI PI AMP</td></tr>
<tr><td></td><td></td><td>W1PRK0</td><td>AmbtrP3A2</td><td>P3A-I</td><td>+</td><td>GGI PI AMP</td></tr>
<tr><td></td><td></td><td>W1PHB1</td><td>AmbtrP3A3</td><td>P3A-I</td><td>+</td><td>GGI PI AMP</td></tr>
<tr><td></td><td></td><td>W1P1C7</td><td>AmbtrP3A4</td><td>P3A-I</td><td>+</td><td>GGI PI AMP</td></tr>
<tr><td></td><td></td><td>XP_011621189</td><td>AmbtrP3A5</td><td>P3A-I</td><td>+</td><td>GGI PI AMP</td></tr>
<tr><td></td><td></td><td>W1NVQ7</td><td>AmbtrP3A6</td><td>P3A-I</td><td>+</td><td>GGI PI AMP</td></tr>
<tr><td></td><td>Arabidopsis thaliana</td><td>P20649</td><td>AtAHA1</td><td>P3A-I</td><td>+</td><td>GGI PI AMP</td></tr>
<tr><td></td><td></td><td>P19456</td><td>AtAHA2</td><td>P3A-I</td><td>+</td><td>GGI PI AMP</td></tr>
<tr><td></td><td></td><td>P20431</td><td>AtAHA3</td><td>P3A-I</td><td>+</td><td>GGI PI AMP</td></tr>
<tr><td></td><td></td><td>Q9SU58</td><td>AtAHA4</td><td>P3A-I</td><td>+</td><td>GGI PI AMP</td></tr>
<tr><td></td><td></td><td>Q9SJB3</td><td>AtAHA5</td><td>P3A-I</td><td>+</td><td>GGI PI AMP</td></tr>
<tr><td></td><td></td><td>Q9SH76</td><td>AtAHA6</td><td>P3A-I</td><td>+</td><td>GGI PI AMP</td></tr>
<tr><td></td><td></td><td>Q9LY32</td><td>AtAHA7</td><td>P3A-I</td><td>+</td><td>GGI PI AMP</td></tr>
<tr><td></td><td></td><td>Q9M2A0</td><td>AtAHA8</td><td>P3A-I</td><td>+</td><td>GGI PI AMP</td></tr>
<tr><td></td><td></td><td>Q42556</td><td>AtAHA9</td><td>P3A-I</td><td>+</td><td>GGI PI AMP</td></tr>
<tr><td></td><td></td><td>Q43128</td><td>AtAHA10</td><td>P3A-I</td><td>+</td><td>GGI PI AMP</td></tr>
<tr><td></td><td></td><td>Q9LV11</td><td>AtAHA11</td><td>P3A-I</td><td>+</td><td>GGI PI AMP</td></tr>
<tr><td></td><td>Oryza sativa japonica</td><td>XP_015632799</td><td>OsAHA1</td><td>P3A-I</td><td>+</td><td>GGI PI AMP</td></tr>
<tr><td></td><td></td><td>XP_015647015</td><td>OsAHA2</td><td>P3A-I</td><td>+</td><td>GGI PI AMP</td></tr>
<tr><td></td><td></td><td>XP_015620234</td><td>OsAHA3</td><td>P3A-I</td><td>+</td><td>GGI PI AMP</td></tr>
</table>

		XP_015638131	OsAHA4	P3A-I	+	GGIPIAMP
		XP_015648645	OsAHA5	P3A-I	+	GGIPIAMP
		XP_015623386	OsAHA6	P3A-I	+	GGIPIAMP
		XP_015635425	OsAHA7a	P3A-I	+	GGIPIAMP
		XP_015628662	OsAHA8a	P3A-I	+	GGIPIAMP
		XP_015632181	OsAHA9	P3A-I	+	GGIPIAMP
		XP_015641851	OsAHA10	P3A-I	+	GGIPIAMP
**SAR**						
**Stramenopiles**						
**Oomyceta**						
	Phytophthora parasitica INRA-310	XP_008901230.1	PhparP3A1	P3A-IIa	-	ASIPIAMQ
	Aphanomyces astaci	XP_009842733.1	AphasP3A1	P3A-I	-	GGIPVAMP
		XP_009842734.1	AphasP3A2	P3A-I	-	GGIPVAMP
	Saprolegnia diclina VS20	XP_008617259.1	SapdiP3A1	P3A-I	-	GGIPVAMP
		XP_008617838.1	SapdiP3A2	P3A-I	-	GGIPVAMP
		XP_008613101.1	SapdiP3A3	P3A-I	-	GGIPVAMP
	Albugo laibachii Nc14	CCA24964.1	AlblaP3A1	P3A-I	-	GGIPVAMP
		CCA15042.1	AlblaP3A2	P3A-IIa	-	ASIPIAMQ
		CCA26167.1	AlblaP3A3	P3A-I	-	GGIPVAMP
	Plasmopara halstedii	XP_024574402.1	PlahaP3A1	P3A-I	-	GGIPVAMP
		XP_024577133.1	PlahaP3A2	P3A-IIa	-	ASIPIAMQ
	Peronospora effusa	RMX68118.1	PerefP3A1	P3A-I	-	GGIPVAMP
		RMX66309.1	PerefP3A2	P3A-IIa	-	ASIPIAMQ
	Bremia lactucae	TDH66910.1	BrelaP3A1	P3A-I	-	GGIPVAMP
		TDH67938.1	BrelaP3A2	P3A-IIa	-	ASIPIAMQ
	Pythium oligandrum	TMW57784.1	PytolP3A1	P3A-I	-	GGIPVAMP
	Achlya hypogyna	OQR99941.1	AchhyP3A1	P3A-I	-	GGIPVAMP
		OQR86857.1	AchhyP3A2	P3A-I	-	GGIPVAMP
		OQS01242.1	AchhyP3A3	P3A-I	-	GGIPVAMP
		OQR86861.1	AchhyP3A4	P3A-I	-	GGIPVAMP
**Ochrophyta**						
	Ochromonadaceae sp. CCMP2298	jgi|Ochro2298_1|332421|e_gw1.2760.3.1	OchroP3A1	P3A-IIa	-	ASIPIAIE
		jgi|Ochro2298_1|344720|estExt_Genewise1.C_6870002	OchroP3A2	P3A-IIa	-	ASIPVAIE
	Thalassiosira pseudonana CCMP1335	XP_002295972.1	ThapsP3A1	P3A-IIa	-	ASIPLAIE
	Phaeodactylum tricornutum CCAP 1055/1	XP_002185425.1	PhatrP3A1	P3A-IId	-	ASIPVALP
		XP_002180325.1	PhatrP3A2	P3A-IIa	-	ASIPLAIE
	Fistulifera solaris	GAX27627.1	FissoP3A1	P3A-IIa	-	ASIPLAIE
	Fragilariopsis cylindrus CCMP1102	OEU16091.1	FracyP3A1	P3A-IId	-	ASIPIALP
	Nannochloropsis salina CCMP1776	TFJ85011.1	NansaP3A1	P3A-IIa	-	ASIPIAIE
		TFJ85012.1	NansaP3A2	P3A-IIa	-	ASIPIAIE
		TFJ80221.1	NansaP3A3	P3A-IIa	-	ASIPIAIE
**Pelagophycea**						
	Aureococcus anophagefferens	XP_009032911.1	AuranP3A1	P3A-IIa	-	ACIPIAMQ
		XP_009038207.1	AuranP3A2	P3A-IIa	-	AAVPVALP
		XP_009039067.1	AuranP3A3	P3A-IId	-	ASIPIALP
	Pelagophyceae sp. CCMP2097	jgi|Pelago2097_1|436352|e_gw1.18.33.1	PelagP3A1	P3A-IIa	-	ASIPIALP
		jgi|Pelago2097_1|531693|fgenesh1_kg.125_#_37_#_Locus9862v1rpkm15.57	PelagP3A2	P3A-IId	-	GAVPIALP
**Labyrinthulomycetes**						
	Hondaea fermentalgiana	GBG30539.1	HonfeP3A1	P3A-I	-	GGIPIAMP
	Aplanochytrium kerguelense PBS07	jgi|Aplke1|49342|fgenesh1_kg.28_#_18_#_isotig04647	AplkeP3A1	P3A-I	-	GGIPIAMP
	Aurantiochytrium limacinum ATCC MYA-1381	jgi|Aurli1|83660|e_gw1.22.29.1	AurliP3A1	P3A-I	-	GGIPIAMP
	Schizochytrium aggregatum ATCC 28209	jgi|Schag1|86033|fgenesh1_kg.56_#_8_#_isotig00663	SchagP3A1	P3A-I	-	GGIPIAMP
**Alveolata**						
**Chromerida**						
	Vitrella brassicaformis CCMP3155	CEL95389.1	VitbrP3A1	P3A-IIb	-	AIVPSAMP
		CEM13152.1	VitbrP3A2	P3A-IIb	-	AVVPIAMP
**Dinophyceae**						
	Symbiodinium microadriaticum	OLP91352.1	SymmiP3A1	P3A-Iid	-	ASVPVALP
**Ciliophora**						
	Stentor coeruleus	OMJ70780.1	StecoP3A1	P3A-IIa	-	ASIPIAMQ
		OMJ72870.1	StecoP3A2	P3A-IIa	-	ASIPIAMQ
		OMJ69746.1	StecoP3A3	P3A-IIa	-	ASIPVAMQ
		OMJ78748.1	StecoP3A4	P3A-IIa	-	ASIPVAMQ
**Apicomplexa**						
	Neospora caninum	XP_003882467.1	NeocaP3A1	P3A-IIb	-	SVAPAAMP
	Toxoplasma gondii ME49	XP_002369362.2	ToxgoP3A1	P3A-IIb	-	SVAPAAMP
**Rhizaria**						
**Cercozoa**						
	Plasmodiophora brassicae	CEP02089.1	PlabrP3A1	P3A-IIa	-	ASIPIAME
		CEO96305.1	PlabrP3A3	P3A-IIa	-	ASIPVAME
		CEO99835.1	PlabrP3A4	P3A-IIa	-	ASIPIAME
**Cryptophyta**						
**Pyrenomonadales**						
	Guillardia theta CCMP2712	XP_005828008.1	GuithP3A1	P3A-IIa	-	ASIPLAIE
		XP_005819019.1	GuithP3A2	P3A-IIa	-	ASIPLAIE
**Haptophyta**						
**Isochrysidales**						
	Emiliania huxleyi CCMP1516	XP_005793796.1	EmihuP3A1	P3A-IId	-	ASVPVALP
		XP_005791681.1	EmihuP3A2	P3A-IIa	-	ASIPIAMQ
**Prymnesiales**						
	Chrysochromulina sp. CCMP291	KOO30309.1	ChrysP3A1	P3A-IIa	-	ASIPIAMQ
		KOO32369.1	ChrysP3A2	P3A-IIa	-	AVVPIANQ
		KOO34192.1	ChrysP3A3	P3A-IIa	-	ASIPIAMQ
**Unikonta**						
**Opisthokonta**						
**Ichthyosporea**						
	Capsaspora owczarzaki ATCC 30864	XP_004344245.1	CapowP3A1	P3A-I	-	GGIPVAMP
	Sphaeroforma arctica JP610	XP_014154558.1	SpharP3A1	P3A-I	-	GGIPIAMP
		XP_014159606.1	SpharP3A2	P3A-I	-	GGIPIAMP
		XP_014146981.1	SpharP3A3	P3A-I	-	GGIPIAMP
		XP_014151490.1	SpharP3A4	P3A-IIa	-	ASIPIAIE
		XP_014156974.1	SpharP3A5	P3A-IIa	-	ASIPIAIE
		XP_014153773.1	SpharP3A6	P3A-IIa	-	ASTPIAIE
**Fungi**						
**Ascomycota**						
**Saccharomycetes**						
	Saccharomyces cerevisiae S288c	P05030	SaccePma1	P3A-IIc	-	IGVPVGLP
		P19657	SaccePma2	P3A-IIc	-	IGVPVGLP
**Eurotiomycetes**						
	Aspergillus nidulans FGSC A4	XP_657922.1	AspniP3A1	P3A-IIc	-	IGVPVGLP
		XP_662463.1	AspniP3A2	P3A-IIc	-	IGVPVGLP
**Leotiomycetes**						
	Pseudogymnoascus pannorum VKM F-3557	KFY01207.1	PsepaP3A1	P3A-IIc	-	IGVPVGLP
		KFX86035.1	PsepaP3A2	P3A-IIc	-	IGVPVGLP
**Sordariomycetes**						
	Fusarium oxysporum Fo47	EWZ49743.1	FusoxP3A1	P3A-IIc	-	VGVPVGLP
		EWZ39463.1	FusoxP3A3	P3A-IIc	-	IGVPVGLP
**Schizosaccharomycetes**						
	Schizosaccharomyces pombe 972h-	NP_594360.1	SchpoPma1	P3A-IIc	-	IGVPVGLP
		NP_587959.2	SchpoPma2	P3A-IIc	-	IGVPVGLP
	Neurospora crassa OR74A	P07038	NeucrPma1	P3A-IIc	-	IGVPVGLP
**Basidiomycota**						
**Agaricomycetes**						
	Agaricus bisporus var. bisporus H97	XP_006463506.1	AgabiP3A1	P3A-I	-	GGIPIAMP
	Coprinopsis cinerea okayama7#130	XP_001832503.1	CopciP3A1	P3A-I	-	GGIPIAMP
		XP_002910572.1	CopciP3A2	P3A-I	-	GGIPIAMP
	Auricularia subglabra TFB-10046 SS5	EJD40156.1	AursuP3A1	P3A-I	-	GGIPIAMP
		EJD43232.1	AursuP3A2	P3A-IIc	-	IGVPVGLP
**Tremellomycetes**						
	Tremella mesenterica DSM 1558	XP_007006406.1	TremeP3A1	P3A-I	-	GGIPIAMP
		XP_007005709.1	TremeP3A2	P3A-IIc	-	IGVPVGLP
**Ustilaginomycetes**						
	Ustilago maydis 521	XP_011387142.1	UstmaP3A1	P3A-I	-	GGIPIAMP
		XP_011388984.1	UstmaP3A2	P3A-IIc	-	VGVPVGLP
	Moesziomyces antarcticus	XP_014657452.1	MoeanP3A1	P3A-IIc	-	VGVPVGLP
		XP_014658969.1	MoeanP3A2	P3A-I	-	GGIPIAMP
**Glomeromycota**						
**Glomeromycetes**						
	Rhizophagus irregularis DAOM 181602=DAOM 197198	XP_025164402.1	RhiirP3A1	P3A-IIc	-	AAIPVGLP
		XP_025189517.1	RhiirP3A2	P3A-IIc	-	AAIPVGLP
**Mucoromycota**						
**Mucoromycotina**						

				Podila verticillata NRRL 6337	KFH68150.1	PodveP3A1	P3A-I	-	GGIPIAMP
					KFH67327.1	PodveP3A2	P3A-IIc	-	AAIPVGLP
					KFH64925.1	PodveP3A3	P3A-IIc	-	AAIPVGLP
					KFH62501.1	PodveP3A4	P3A-IIc	-	AAIPVGLP
					KFH66528.1	PodveP3A5	P3A-IIc	-	AAIPVGLP
				Mucor circinelloides f. circinelloides 1006PhL	EPB81160.1	MucciP3A1	P3A-I	-	GGIPIAMP
					EPB91649.1	MucciP3A2	P3A-I	-	GGIPIAMP
					EPB90213.1	MucciP3A3	P3A-IIc	-	AAIPVGLP
					EPB87345.1	MucciP3A4	P3A-IIc	-	AAIPVGLP
					EPB89056.1	MucciP3A5	P3A-IIc	-	AAIPVGLP
				Rhizopus delemar RA 99-880	EIE78929.1	RhideP3A1	P3A-I	-	GGIPIAMP
					EIE87190.1	RhideP3A2	P3A-I	-	GGIPIAMP
					EIE81463.1	RhideP3A3	P3A-IIc	-	AAIPVGLP
					EIE89187.1	RhideP3A4	P3A-IIc	-	AAIPVGLP
					EIE90472.1	RhideP3A5	P3A-IIc	-	AAIPVGLP
				Rhizopus microsporus ATCC 52813	XP_023466301.1	RhimiP3A1	P3A-IIc	-	AAIPVGLP
					XP_023466973.1	RhimiP3A2	P3A-IIc	-	AAIPVGLP
					XP_023468080.1	RhimiP3A3	P3A-IIc	-	AAIPVGLP
					XP_023471391.1	RhimiP3A4	P3A-I	-	GGIPIAMP
			**Mortierellomycotina**						
				Lobosporangium transversale	XP_021885117.1	LobtrP3A1	P3A-IIc	-	AAIPVGLP
					XP_021885431.1	LobtrP3A2	P3A-IIc	-	AAIPVGLP
					XP_021877379.1	LobtrP3A3	P3A-IIc	-	AAIPVGLP
					XP_021878950.1	LobtrP3A4	P3A-IIc	-	AAIPVGLP
					XP_021880437.1	LobtrP3A5	P3A-IIc	-	AAIPVGLP
					XP_021880984.1	LobtrP3A6	P3A-IIc	-	AAIPVGLP
					XP_021884630.1	LobtrP3A7	P3A-I	-	GGIPIAMP
		**Zoopagomycota**							
			**Zoopagomycotina**						
				Syncephalis pseudoplumigaleata	RKP27234.1	SynpsP3A1	P3A-IIc	-	SAVPVALP
			**Entomophthoromycotina**						
				Basidiobolus meristosporus CBS 931.73	ORX92534.1	BasmeP3A1	P3A-IIc	-	AAIPVGLP
					ORY05772.1	BasmeP3A2	P3A-IIc	-	AAIPVGLP
					ORY05773.1	BasmeP3A3	P3A-IIc	-	AAIPVGLP
					ORX81304.1	BasmeP3A4	P3A-IIc	-	IGVPVGLP
					ORX92659.1	BasmeP3A5	P3A-IIc	-	IGVPVGLP
					ORX64691.1	BasmeP3A6	P3A-IIc	-	VGVPVGLP
					ORX92664.1	BasmeP3A7	P3A-IIc	-	VGVPVGLP
					ORX92665.1	BasmeP3A8	P3A-IIc	-	VGVPVGLP
					ORX92666.1	BasmeP3A9	P3A-IIc	-	VGVPVGLP
			**Kickxellomycotina**						
				Dimargaris cristalligena	RKP39731.1	DimcrP3A1	P3A-I	-	GGIPIAMP
					RKP39492.1	DimcrP3A2	P3A-IIc	-	AAIPVGLP
		**Blastocladiomycota**							
			**Blastocladiomycetes**						
				Allomyces macrogynus ATCC 38327	KNE65274.1	AllmaP3A1	P3A-IIc	-	AAIPVGLP
		**Chytridiomycota**							
			**Chytridiomycetes**						
				Spizellomyces punctatus DAOM BR117	XP_016610781.1	SpipuP3A1	P3A-IIc	-	IGVPVGLP
					XP_016611460.1	SpipuP3A2	P3A-IIc	-	AAIPVGLP
	**Rotosphaerida**								
				Fonticula alba	XP_009495892.1	FonalP3A1	P3A-I	-	GGIPIAMP
	**Apusozoa**								
			**Apusomonadidae**						
				Thecamonas trahens A TCC 50062	XP_013753677.1	ThetrP3A1		-	ASIPIALP
	**Amoebozoa**				XP_013759271.1	ThetrP3A2		-	AAIPVAMP
			**Discosea**						
				Acanthamoeba castellanii str. Neff	XP_004333016.1	AcacaP3A1	P3A-I	-	GGLPIAMP
			**Eumycetozoa**						
				Heterostelium album PN500	XP_020436662.1	HetalP3A1	P3A-I	-	GGIPIAMP
				Acytostelium subglobosum LB1	XP_012757040.1	AcysuP3A1	P3A-I	-	GGIPIAMP
					XP_012753723.1	AcysuP3A2	P3A-I	-	GGIPIAMP
					XP_012759216.1	AcysuP3A3	P3A-I	-	GGIPIAMP
				Cavenderia fasciculata	XP_004361006.1	CavfaP3A1	P3A-I	-	GGIPIAMP
				Dictyostelium purpureum	XP_003284057.1	DicpuP3A1	P3A-I	-	GGIPIAMP
				Dictyostelium discoideum AX4	XP_639363.1	DicdiPMA1	P3A-I	-	GGIPIAMP
				Dictyostelium fasciculatum	XP_004361006.1	DicfaPMA1	P3A-I	-	GGIPIAMP
				Polysphondylium pallidum PN500	EFA84549.1	PolpaPMA1	P3A-I	-	GGIPIAMP
				Acytostelium subglobosum LB1	XP_012757040.1	AcysuP3A1	P3A-I	-	GGIPIAMP
					XP_012753723.1	AcysuP3A2	P3A-I	-	GGIPIAMP
					XP_012759216.1	AcysuP3A3	P3A-I	-	GGIPIAMP
	**Discoba**								
			**Euglenozoa**						
				Bodo saltans	CUF89536.1	BodsaP3A1	P3A-IIa	-	VSIPIAIE
				Strigomonas culicis	EPY37061.1	StrcuP3A1	P3A-IIa	-	VSIPIALE
				Trypanosoma cruzi	XP_812631.1	TrycrP3A1	P3A-IIa	-	VSIPIALE
				Leishmania major strain Friedlin	XP_001682560.1	LeimaPMA1	P3A-IIa	-	VSIPIALE
**Archaea**									
		**Euryarchaeota**							
			**Methanobacteria**						
				Methanobacterium paludis	WP_048187944.1	MetpaP3A1		-	ASIPVALP
					WP_013825693.1	MetpaP3A2		-	ASIPVAQP
			**Methanococci**						
				Methanocaldococcus bathoardescens	WP_048201651.1	MetbaP3A1		-	SAIPAAMP
				Methanothermococcus okinawensis IH1	AEH06486.1	MetokP3A1		-	ASIPAAMP
			**Methanomicrobia**						
				Methanosarcina acetivorans C2A	AAM05085.1	MetacP3A1		-	AAIPAALP
					AAM06212.1	MetacP3A2		-	AAIPAALP
				Methanococcoides burtonii DSM 6242	ABE51089.1	MetbuP3A1		-	AAIPAALP
				Methanolobus vulcani	SDG28837.1	MetvuP3A1		-	AAIPAALP
			**Thermoplasmata**						
				Aciduliprofundum boonei T469	ADD08967.1	AciboP3A1		-	AAIPAALP
**Eubacteria**									
			**Alphaproteobacteria**						
				Methyloceanibacter caenitepidi	WP_082025528.1	MetcaP3A1		-	AAIPVAMP
				Rhodopseudomonas palustris	WP_044414997.1	RhopaP3A1		-	ASIPVALP
					WP_110786469.1	RhopaP3A2		-	ASIPVALP
			**Gammaproteobacteria**						
				Psychromonas arctica	WP_028870080.1	PsyarP3A1		-	AAIPVAMP
				Methylomarinum vadi	WP_051906753.1	MetvaP3A1		-	ASIPVAMP
			**Deltaproteobacteria**						
				Geobacter metallireducens RCH3	EHP85803.1	GeomeP3A1		-	AAIPVAMP
				Desulfomonile tiedjei	WP_014809784.1	DestiP3A1		-	AAIPVAMP
			**Acidithiobacillia**						
				Acidithiobacillus ferrooxidans	WP_215854205.1	AcifeP3A1		-	ASIPVAMP
					WP_163059761.1	AcifeP3A2		-	ASIPVAMP
			**Actinobacteria**						
				Mycobacterium bohemicum DSM 44277	CPR07252.1	MycboP3A1		-	AAIPVAMP
					CPR05526.1	MycboP3A2		-	ASIPVALP
				Mycolicibacterium aromaticivorans	WP_051660314.1	MycarP3A1		-	AAIPVAMP
					WP_036343692.1	MycarP3A2		-	ASIPVALP
			**Cyanobacteria**						
				Phormidesmis priestleyi	WP_068818899.1	PhoprP3A1		-	ASIPVAMP
				Cyanobacterium aponinum PCC 10605	AFZ52990.1	CyaapP3A1		-	ASIPVAMP
				Crocosphaera subtropica	WP_009546079.1	CrosuP3A1		-	ASIPLATP
			**Planctomycetes**						
				Rhodopirellula sallentina SM41	EMI58256.1	RhosaP3A1		-	AAIPVAMP

Supplemental Table 2: Primer sequences

	Primer name	Direction	Sequence (5′–3′)
sgRNAs	sgRNA for aha2Δ30	F	GTT ATA TGT TAA ACA GGC TT
	sgRNA for aha2Δ44	F	GTT AAC ATC TTC CCT GAG AA
	sgRNA for aha2Δ57	F	GAG AAG AGA GAG AGG CTC AAT
Genotyping	Genotyping F	F	GAT TGC GGT TTA CGC CAA CT
	Genotyping R	R	CCA AAT GCC AAA CAC AAA GGC
	Sequencing	F	CCA AGC CCT TTA GCT TCA CG
