# Supplementary material for: The evolution of plant proton pump regulation via the R domain may have facilitated plant terrestrialization
Source: Commun Biol. 2022 Nov 29;5:1312. doi: 10.1038/s42003-022-04291-y (PMC9708826; doi:10.1038/s42003-022-04291-y)
Supplement: Supplementary file 2 — Description of Additional Supplementary Data [file 42003_2022_4291_MOESM2_ESM.docx]

**Description of Additional Supplementary Files**

**File name:** Supplementary Data 1

**Description:** The numerical source data for graphs presented in Figures 3, 4, 5, 6, 8.
